# Evaluation of the anticancer activity of RIN-1, a Notch signaling modulator, in head and neck squamous cell carcinoma

**DOI:** 10.1038/s41598-023-39472-0

**Published:** 2023-08-22

**Authors:** Arkadiusz Czerwonka, Joanna Kałafut, Shaoxia Wang, Alinda Anameric, Alicja Przybyszewska-Podstawka, Jesse Mattsson, Mahtab Karbasian, Doriane Le Manach, Mervi Toriseva, Matthias Nees

**Affiliations:** 1 Department of Biochemistry and Molecular Biology, Medical University of Lublin, 20-093 Lublin, Poland; 2 FICAN West Cancer Centre Laboratory, Cancer Research Unit, Institute of Biomedicine, Turku University Hospital, University of Turku, Turku, Finland

**Keywords:** Head and neck cancer, Oral cancer

## Abstract

Notch signalling is one of the key molecular pathways involved in cell-to-cell signal transduction. Although the mechanisms of action of the NOTCH receptors are already relatively well known, their biological implications remain unclear, especially during the initiation and progression of head and neck squamous cell carcinoma (HNSCC). Here, we present the growth- and differentiation-modulating effects of various “next generation” small molecule Notch modulators represented by RIN-1, and CB-103, on HNSCC, compared to gamma secretase inhibitors as “conventional” NOTCH interfering compounds, like DAPT. These molecules were tested in different cell- and tissue culture conditions represented by 2D monolayer, non-adherent or spheroid culture, 3D organoid cultures, and zebrafish in vivo model. The most pronounced, pleiotropic effects were observed for the NOTCH modulator RIN-1. At the molecular level, RIN-1-dependent activation of Notch signalling led to characteristic changes in the expression of NOTCH-regulated targets, i.e., the transcriptional suppressors HES1 and HEY1, p21 (CDKN1A) cell cycle inhibitor, and pro-apoptotic BAX markers. These changes led to restriction of proliferation, growth, and reduced motility of HNSCC cells in 2D cultures. Consequently, cell cycle arrest in the G2-M phase and induction of apoptosis were observed. Similar anticancer effects were observed in 3D cultures and in the zebrafish model. In contrast, RIN-1 treatment resulted in inhibition of Notch signalling and the growth of HNSCC spheroids under non-adherent cell culture conditions. Our results suggest that modulation of Notch signalling could be used as a chemotherapeutic agent in selected patients with intact NOTCH signaling.

## Introduction

**Figure 1:**
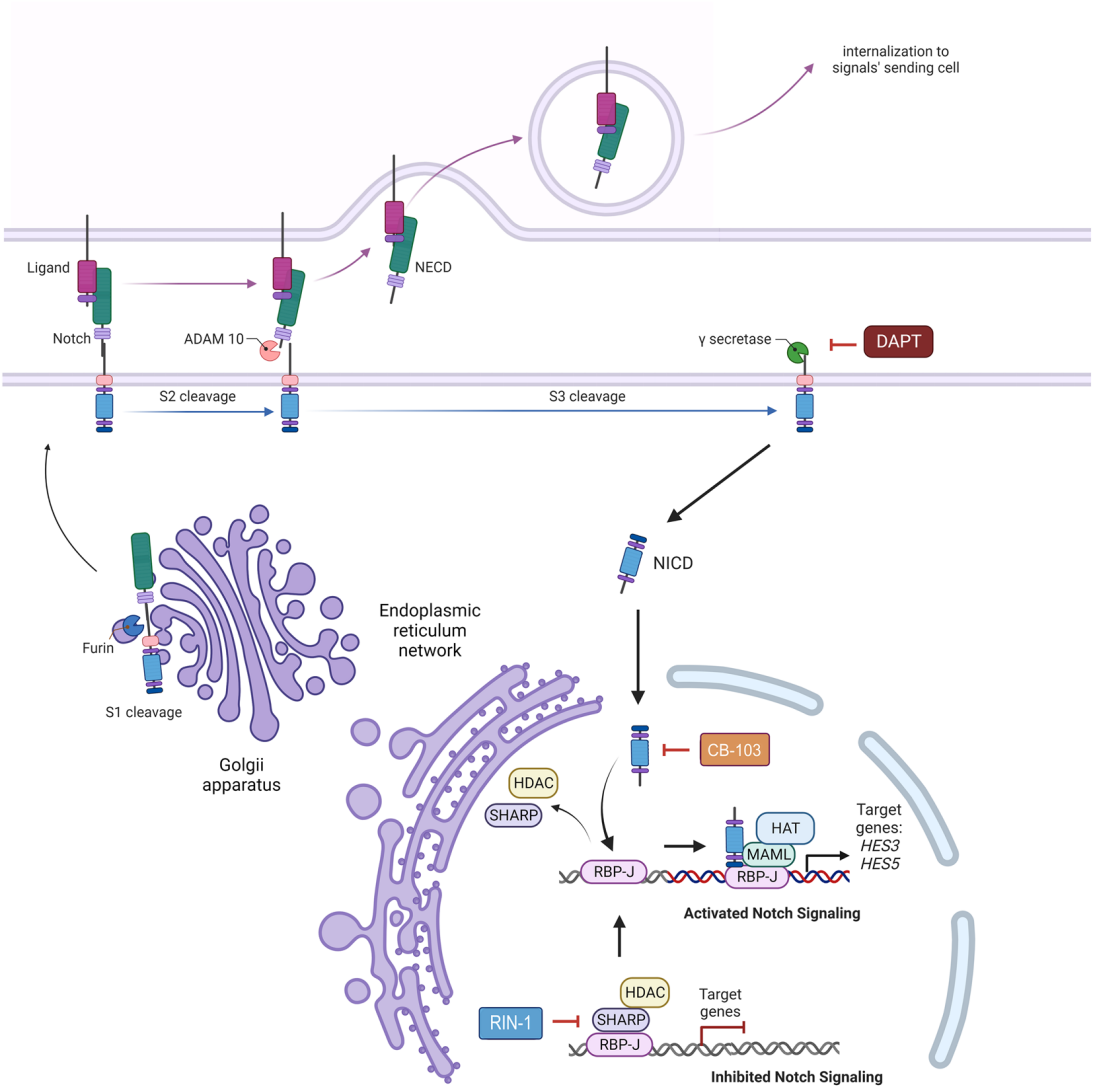
The Notch signalling pathway and its modulators used in the present study. NOTCH proteins undergo maturation and post-translational processing within the Endoplasmic Reticulum (ER) and Golgi apparatus, prior to its transfer to the plasma membrane. The four membrane-anchored Notch receptors, presented on the surface of the signal-receiving cells, interact with any of the five ligands, presented by the signal-sending cell (trans-signalling). Signal-receiving and sending cells may also be identical (cis-signalling). This interaction results in a series of 3 proteolytic cleavages that eventually lead to release of the active Notch intracellular domain (NICD) into the cytoplasm. After translocation to the nucleus, the NICD of all four Notch receptors binds to CSL/RBP-J and further interacts with transcriptional coactivators, thus forming a transcriptional activator/repressor complex that regulates the expression of cell-type specific downstream target genes. RBP-J is also involved in the formation of transcriptional repressor complexes through binding to SHARP and HDAC. The Notch signalling modulators, **RIN-1**, **CB-103,** and **DAPT** and their known molecular targets are indicated in the figure.

As transmembrane mechanoreceptors, NOTCH receptors are involved in signal transduction between different cell types in early embryogenesis, neural and vascular tissue development, and play a pivotal role in the maintenance of post-natal tissue homeostasis^1^. Notch signalling involves at least one of the four NOTCH mechanoreceptors, NOTCH1-4, and one of their ligands, such as Delta-like 1, 3 or 4 (DLL1; 3; 4), or Jagged 1 and 2 (JAG1 and JAG2). Upon ligand activation, the NOTCH receptors undergo a series of three proteolytic events, culminating in γ-secretase cleavage and release of the Notch intracellular domain (NICD), which translocates to the nucleus to regulate cellular gene expression. In the nucleus, the NICD acts by binding to RBP-J (Recombination signal binding protein for immunoglobulin kappa J region; known also as CSL (CBF-1/suppressor of hairless/Lag1)), supported by MAML1-3 (Mastermind-like transcriptional coactivators 1–3), and histone acetyltransferases (HATs, such as p300, CBP, and P/CAF) that act as co-repressors or co-activators. In this fashion, the NICD represents an active transcriptional complex that regulates the expression of a largely cell-type specific panel of downstream genes, including members of the HES and HEY family transcriptional regulator proteins, such as *HES1-6* (Hes family bHLH transcription factors 1–6), and *HEY1* (Hes related family bHLH transcription factor with YRPW motif 1)^2^. These proteins mainly act as transcriptional repressors^3^. The CSL/RBP-J is also a crucial element of the SHARP-dependent transcription repressor complex, related to the activity of histone deacetylases (HDAC), and consequently, epigenetic silencing events. In the absence of Notch signalling, RBP-J interacts with a SHARP scaffold protein with allows the formation of a repression complex which acts via HDAC activity^4^. Despite the relative similarities in the structure of four Notch receptors, their activation, and signal transmission, the final effect of Notch pathway activation is at least partially specific for each of them, and also significantly different between various cell- and tissue types; which ensures high plasticity in the control of gene expression^5^. Notch signalling is related to controlling a broad spectrum of pivotal cell fate decisions and is involved in cancer-related processes such as cell growth and proliferation, tissue- or lineage-specific differentiation, maintenance of stemness, changes in cellular metabolism, and cell viability. Therefore, aberrations in the Notch pathway are often associated with the initiation and progression of cancers, including head and neck squamous cell carcinoma (HNSCC)^5–7^. In different tumour entities, however, the role of Notch signalling in cancer initiation and progression may be markedly different or even opposing—especially between hematopoietic and epithelial cancers. Oncogenic, activating, or gain-of-function (GoF) NOTCH1 mutations and hyper-activated Notch signalling are primarily reported in T-ALL and triple-negative breast cancers (TNBC). In contrast, data on the molecular effects of perturbed Notch signalling in HNSCC and other squamous cell carcinomas (SCC) indicate that NOTCH receptors and their ligands act as potent tumour suppressors, with characteristic loss-of-function (LoF) mutations. The same LoF mutations are also found in healthy, but genetically damaged tissues such as sun-exposed skin^8^, and are considered gatekeeper mutations that may initiate or promote early stages of cancer development. At later stages of cancer progression, especially in acquired chemo- and radioresistance, tumour relapse, local or distant metastasis, and invasion, NOTCH signalling may play a dual role in tumour development. This re-activation or hyper-activation depends strongly on the nature of the specific cell type, cell–cell- and ligand-receptor interactions, and the functions of signal receiving and signal transmitting cells. Thus, the tumour suppressor function with LoF alterations may only be a common feature in early-stage tumour development^6,9–11^, but continued wildtype-level and hyperactivated Notch signalling may then (re-)activate and support later stage cancer progression of HNSCC. This is currently poorly understood, partly because there is a lack of research centred on late stage, recurrent/metastatic HNSCC and drug resistance tumours, the redundancy of the signalling pathways, and also because most advanced (and metastatic) tumours are not routinely operated and therefore are missing for translational research. For this project, we aimed to test a panel of small molecule chemical modulators of NOTCH signalling, in addition to gamma secretase inhibitors (GSI) as conventional NOTCH inhibitors. Here, we present data demonstrating the diverse effects of several next generation Notch signalling-specific drugs (Notch modulators, Fig. 1), with a focus on **RIN-1** (an RBPJ inhibitor blocking the functional interaction of RBP-J with SHARP^12^), considered a Notch signalling activator). We also tested **CB-103**, a pan-NICD inhibitor blocking the functional interaction of RBPJ with NICD^13^). As a reference compound, we have also used one GSI in our studies (DAPT), which blocks the cleavage of all four full-length Notch proteins and prohibits intracellular and nuclear translocation of the NICD.

It remains unclear if any of the compounds mentioned above are in any way specific or selective for certain Notch receptors, or if they represent pan-specific Notch inhibitors, as claimed for some. Here, we are using a panel of cancer cell lines established from HNSCC biopsies in the 1990s^14^, which are also available as very early passages. We selected 4 lines without any apparent Notch receptor or downstream modulator mutations like AJUBA; EP300, or FBXW7, from a panel of 45 lines with known NOTCH signalling activity and mutation status^14^, and validated expression of Notch receptors 1, 2, and 3. NOTCH 4 was not further analysed since we noticed very low to missing expression, in line with its specific expression in endothelial cells. All lines of the UTSCC panel are derived from primary HNSCC tumours (A-lines) or recurrent/metastatic lesions (B-lines), and serve as physiologically relevant models for tumours with a broad spectrum of differential Notch signalling pathway activity. Physiological or wild-type level NOTCH signalling in HNSCC in this study is represented by the cell lines UT-SCC-24A (primary tongue carcinoma), UT-SCC-24B (obtained from a recurrent site of the same patient), UT-SCC-42A (primary larynx tumour) and UT-SCC-42B (obtained from a cervical lymph node metastasis of the same patient). The impact of Notch signalling modulators on HNSCC cell growth was first assessed in two-dimensional (2D) monolayer cultures, and subsequently validated in non-adherent spheroid conditions and three-dimensional (3D) “organotypic” or organoid cultures, followed by in vivo cultures using the zebrafish (*Danio rerio)* model.

## Materials and methods

### Cell lines and cell culture conditions

The University of Turku-Squamous Cell Carcinoma (UT-SCC) cell lines UT-SCC-24A (Tongue squamous cell carcinoma), UT-SCC-24B (Tongue squamous cell carcinoma, derived from metastatic site: cervical lymph node) UT-SCC-42A (Laryngeal squamous cell carcinoma) and UT-SCC-42B (Laryngeal squamous cell carcinoma, derived from metastatic site: cervical lymph node) were all established and obtained from Turku University Central Hospital, Finland; courtesy of Prof. Reidar Grenman. Normal human foreskin fibroblasts BJ (ATCC nub. CRL-2522), hTERT-immortalized human PF179T cancer-associated fibroblasts (CAF, ATCC nub. CRL-3290) and MCF-7 (human breast adenocarcinoma) cell lines were obtained from the biobank of the Institute of Biomedicine, Cancer Research Unit and FICAN West Cancer Centre Laboratory, University of Turku and Turku University Hospital. HEK (Human epithelial-like kidney cells) 293 T cell line was obtained from ATCC (ATCC nub. CRL-3216). Cells were cultured in DMEM-F12 (Sigma-Aldrich) or DMEM, GlutaMAX (Gibco) medium supplemented with 10% FBS and penicillin (100 units/ml) / streptomycin (100 µg/ml) antibiotics at standard culturing condition (37 °C, 5% CO_2_). To determine the proper cell density, cells were counted using a TC20™ Automated Cell Counter (Bio-Rad) before each experiment.

### Notch signalling modulators and other chemical reagents

RIN-1 (2-(2-Fluorophenoxy)-4-(1-methyl-1H-pyrazol-5-yl)benzamide)) and CB-103 (6-(4-Tert-butylphenoxy)pyridin-3-amine)) were purchased from Selleck Chemicals GmbH (Munich, Germany). DAPT [N-[N-(3,5-Difluorophenacetyl)-L-alanyl]-S-phenyl-glycine t-butyl ester] was purchased from Merck. Compounds' Stock solutions (50 mM) were prepared in DMSO according to the manufacturer's instruction and stored at −80 °C. All other consumables were obtained from Merck (Darmstadt, Germany) unless indicated differently.

### Cell viability, proliferation and growth assays in 2D condition

For MTT viability and proliferation analyses tested cells were seeded onto a 96-well plate at a density of 3–5 × 10^4^ cells/mL. After 24 h, the culture medium was removed and the cells were exposed to serial dilutions of the tested compounds. Cells were incubated through 96 h and exposed to 15 µl MTT (3-(4,5-dimethylthiazol-2-yl) -2,5-diphenyltetrazolium bromide, 5 mg/ml of PBS). After 3 h, formed formazan crystals were solubilized by adding SDS buffer (10% SDS in 0.01 N HCl, overnight incubation). Absorbance was determined at a wavelength of 570 nm using an M200 Pro microplate reader (Tecan). For cell growth assay in 2D condition, cells were seeded onto a 96-well plate at a density of 3–5 × 10^4^ cells/mL. After 24 h the culture medium was removed and the cells were exposed to serial dilutions of RIN-1. Cells were imaged every 2 h for 84 h with an Incucyte (Essen BioScience, Royston Hertfordshire, UK) in standard culturing conditions. Cells’ growth was calculated based on cells’ confluence by the Incucyte software tool (Sartorius Stedim Biotech, Goettingen, Germany).

### Luciferase reporter assay

To measure NICD activity, a Firefly Luciferase (12xCSL-Luc) reporter system^15,16^ was used. The Notch 12xCSL-Luc activity reporter contains high affinity CBF1/Suppressor of Hairless/Lag 1 (CSL) binding sites (12 × CGTGGGAA), linked to the Firefly Luciferase gene^17^. The formation of a CSL/NICD complex leads to the activation of transcription and the presence of luciferase in the cell. The HEK-293 cells were transfected with 12xCSL-Luc plasmid using TurboFect and selected with geneticin (G418) to obtain a stabile line^18^. The HEK293 cells with stable expressed 12xCSL-Luc reporter were seeded onto a two 24-well plate at a density of 5 × 10^4^ cells/mL. Next day one plate was transfected by the ΔE NICD1^19^ using Turbofect™ transfection reagent (following the manufacturer’s protocol). ΔE NICD1 is a membrane-tethered, constitutively active construct whose activation is independent of the presence of a ligand but remains sensitive to the action of γ-secretase. Second plate was transfected by empty plasmid (mock transfection). After 24 h, the HEK293 cells were treated with proper concentrations of Notch modulators. After the next 48 h, 12xCSL-Luc transfected HEK293 cells were harvested and lysed following to manufacturer’s protocol (Bright-Glo Luciferase Assay System, Promega). The equal volume of lysates and Bright-Glo Luciferase reagent was transferred to a black microplate well and measured using a microplate luminometer (Tecan Infinite 200 PRO). The relative luminescence units (RLU) level were normalized to the cell number count in each sample.

### Wound assay

For wound assay cells were seeded onto a 96-well plate at a full confluence (density of 5 × 10^5^ cells/mL). After cell adhesion, scratch wounds were generated with the Incucyte Woundmaker 96-Tool. Next cells were exposed to serial dilutions of the tested compounds and imaged every 1–2 h until full confluence and wound closure was obtained (14-24 h). Cell migration was assessed based on the rate of cell migration into the wound area (wound with; μm) by using the Incucyte software tools.

### LDH cell toxicity assay

For LDH toxicity assay, the CyQUANT™ LDH Cytotoxicity Assay kit (Thermo Fisher) was used. The tested cell lines were seeded on 96-well plates (5 × 10^5^ cells/ml) in 100 μl of culture medium. After 24 h cells were exposed to different concentrations of the tested compounds in cell culture medium, supplemented with 2% FBS. After 48 h of incubation, the supernatants were collected and an LDH toxicity assay was performed according to the manufacturer's instructions. Absorbance was determined at a wavelength of 570 nm using an M200 Pro microplate reader (Tecan).

### Apoptosis assessment

For both Annexin V/Propidium iodide (PI) and active caspase-3 assays, the tested cells were seeded onto 6-well microplates at a density of 5 × 10^5^ cells/mL. The next day, the culture medium was removed, and the cells were washed with PBS (Ca^2+^ and Mg^2+^) and exposed to the tested compounds. After 48 h of incubation cells were washed with PBS (without Ca^2+^ and Mg^2+^) and harvested (5 mM EDTA in PBS without Ca^2+^ and Mg^2+^). After detaching, cells were centrifuged (500×*g*), washed in PBS, and further analysed in flow cytometer. The number of apoptotic and necrotic cells was measured with the FITC Annexin V Apoptosis Detection Kit I (BD Pharmingen). Additionally, the activation of caspase-3 was measured by PE Active Caspase-3 Apoptosis Kit (BD Pharmingen). Both assays were performed according to the manufacturer's instructions. The samples were analysed using flow cytometry (BD FACSCalibur, CellIst Pro Version 6.0. software for the Macintosh operating system) directly after preparation. For each analysis, 10,000 events were acquired.

### Growth of HNSCC cells in non-adherent conditions

Non-adherent conditions was obtained by culturing HNSCC cells in 96-well U-bottom low attachment microplate (Corning). In brief, cells were seeded at a density of 4000 cells/well, and immediately treated by 2.5 µM of RIN-1, CB-103 and DAPT, respectively (n ≥ 3). Next, cells were incubated at 37 °C for the duration of the experiment (168 h). Organoids photos were obtained by using EVOS M5000 Imaging System (Thermo Fisher Scientific). Further organoids shape and area measuring were processed by using ImageJ-Fiji (www.imagej.net) software. Material for qPCR experiments was obtained in non-adherent conditions, organoids from at least 15 wells (n ≥ 15) were collected.

### 3D organoids experiments

All 3D organoids cultures were prepared as a “sandwich model” in 96-well Angiogenesis plates (Ibidi GmbH, Munich, Germany), and Matrigel Growth Factor Reduced (GFR) Basement Membrane Matrix (Corning). In brief, the bottom of Angiogenesis μ-slides wells was filled with 10 µl of 50% Matrigel in medium (typically 3–5 mg/ml protein, depending on the batch), and incubated at 37 °C for 30–60 min. Cells were placed on top of the polymerized bottom gel at a density of 2000 cells/well, and incubated at 37 °C for 1–2 h. The medium was discarded, and cell layers were covered with 20 µl of 25% Matrigel (1.5–2.5 mg/ml depending on the batch). The μ-slides were humidified by adding sterile water to the reservoirs at the plate’s outer edges. The upper gel was allowed to polymerize at 37 °C for 3–4 h or overnight. For 3D cell proliferation experiments, the cells were treated immediately after seeding, using medium containing a proper concentration of Notch modulators. For experiments with Notch modulators on more mature HNSCC organoids, cells were left to grow and form organoids for 3 days and exposed to an appropriate concentration of Notch modulators. After that experiments were continued for an additional 3 days. During experiments, 3D organoids were observed by real-time visualization in Incucyte live-cell imager (Essen Bioscience). Control, as well as Notch modulators-containing medium, were changed every second day.

### 3D cell viability assay

After 3D organoids experiments the WST-8 cell viability assay was performed by using Cell Counting Kit-8 (WST-8/CCK8, DojinDO Laboratories, Japan) according to the manufacturer protocol. Absorbance was measured at a wavelength of 450 nm with a plate reader (WALLAC VICTOR^2^).

### Quantitative PCR (qPCR) analysis

For qPCR analysis, cells were harvested (5 mM ETDA in PBS without Ca^2+^ and Mg^2+^) and centrifuged (500x*g*) and total RNA was extracted using Extract Total RNA kit (Blirt) according to the manufacturer protocol. Next, the isolated total RNA was used as a template for cDNA synthesis through High-Capacity cDNA Reverse Transcription Kit (Applied Biosystems). The qPCR analysis was performed with the LightCycler 480 II instrument (Roche) using PowerUp SYBR Green Master Mix (Applied Biosystems), specific forward/reverse primers pairs and 25 ng of cDNA in each reaction. The number of cycles needed to reach a specific threshold of detection (CT) was used to calculate the relative quantification. Relative mRNA expression was calculated using the 2^-∆∆Ct^ subtraction method, and normalized to the expression of the *GAPDH* housekeeping gene. FC values in the range of 0–0.749; 0.75–1.5; 1.501–10 were considered as downregulated, no change, or upregulated, respectively, relative to target gene expression.

Primer sequences (forward 5'–3' and reverse 3'–5') for individual genes were obtained from the OriGene database (https://www.origene.com) or artificially designed (Benchling software) based on Ensembl Genomes database and then synthesized (GenoMed). All primers used for qPCR were tested for specificity and sensitivity. The following sequences were used in the research:

*GAPDH* (forward 5’-GTGGAGTCTACTGGTGTCTTC-3’, revers 3’-GTGCAGGAGGCATTGCTTACA-5’),

*NOTCH1* (forward 5’-CAACTGCCAGAACCTTGTGC-3’ and reverse 3'-GGCAACGTCAACACCTTGTC-5'),

*NOTCH2* (forward 5'-GGCACGTCAGGGGTTAATTG-3' and reverse 3'- GCGGAAACCATTCACACCGTTGAT-5'),

*NOTCH3* (forward 5'-GCAGATGGCTCAACGGCACTG-3' and reverse 3'-GGGGTCTCCTCCTTGCTATCCTG-5'),

*NOTCH4* (forward 5'-GAGGACAGCATTGGTCTCAAGG-3' and reverse 3'-TGTCACCCCATCAGGTCCAC-5'),

*JAG1* (forward 5'-GCCGAGGTCCTATACGTTGC-3' and reverse 3'-CCGAGTGAGAAGCCTTTTCAA-5'),

*JAG2* (forward 5'-GCTGCTACGACCTGGTCAATGA-3' and reverse 3'-AGGTGTAGGCATCGCACTGGAA-5'),

*DLL1* (forward 5'-TGCCTGGATGTGATGAGCAGCA-3' and reverse 3'-ACAGCCTGGATAGCGGATACAC-5'),

*DLL3* (forward 5'-CACTCAACAACCTAAGGACGCAG-3' and reverse 3'-GAGCGTAGATGGAAGGAGCAGA-5'),

*DLL4* (forward 5'-CTGCGAGAAGAAAGTGGACAGG-3' and reverse 3'-ACAGTCGCTGACGTGGAGTTCA-5'),

*ADAM17* (forward 5'-AACAGCGACTGCACGTTGAAGG-3' and reverse 3'-CTGTGCAGTAGGACACGCCTTT-5'),

*ADAM10* (forward 5'-GAGGAGTGTACGTGTGCCAGTT-3' and reverse 3'-GACCACTGAAGTGCCTACTCCA-5'),

*MAML1* (forward 5'-GCAACAGCAGTTCCTTCAGAGG-3' and reverse 3'-GTGAACTGTCCAACCTGCTGTG-5'),

*MAML2* (forward 5'-GGTCACCTTTGCCACTTCAGCA-3' and reverse 3'-AGCAGGGGTTAGGACTTGGACT-5'),

*MAML3* (forward 5'-CACAGCGGAATCCATACCCAGT-3' and reverse 3'-ATGCCTGCGTTCTGTGCCATCA-5'),

*R-BPJ* (CSL; forward 5'-TCATGCCAGTTCACAGCAGTGG-3' and reverse 3'-TGGATGTAGCCATCTCGGACTG-5'),

*DVL3* (forward 5'-GTGACCGCATGTGGCTCAAGAT-3' and reverse 3'-CGTGAAGCCTTCCACATTGTGG-5'),

*HES1* (forward 5'-TCAACACGACACCGGATAAAC-3' and reverse 3'-GCCGCGAGCTATCTTTCTTCA-5'),

*HEY1* (forward 5'-CGGCTCTAGGTTCCATGTCC-3' and reverse 3'-GCTTAGCAGATCCCTGCTTCT-5'),

*BAX* (forward 5'-TCAGGATGCGTCCACCAAGAAG-3' and reverse 3'-TGTGTCCACGGCGGCAATCATC-5'),

*BCL-2* (forward 5'-ATCGCCCTGTGGATGACTGAGT-3' and reverse 3'-GCCAGGAGAAATCAAACAGAGGC-5'),

*CDKN1Am*(p21; forward 5'-AGGTGGACCTGGAGACTCTCAG-3' reverse 3'-TCCTCTTGGAGAAGATCAGCCG-5'),

*CDKN1B* (p27; forward 5'-ATAAGGAAGCGACCTGCAACCG-3' reverse 3'-TTCTTGGGCGTCTGCTCCACAG-5'),

*MKI67* (Ki-67; forward 5'-GAAAGAGTGGCAACCTGCCTTC-3' reverse 3'-GCACCAAGTTTTACTACATCTGCC-5'),

*TOP2A* (forward 5'-GTGGCAAGGATTCTGCTAGTCC-3' and reverse 3'-ACCATTCAGGCTCAACACGCTG-5'),

*CCNA2* (forward 5'-CTCTACACAGTCACGGGACAAAG-3' and reverse 3'-CTGTGGTGCTTTGAGGTAGGTC-5'),

*CCNB1* (forward 5'-GACCTGTGTCAGGCTTTCTCTG-3' and reverse 3'-GGTATTTTGGTCTGACTGCTTGC-5'),

*CCNE1* (forward 5'-TGTGTCCTGGATGTTGACTGCC-3' and reverse 3'-CTCTATGTCGCACCACTGATACC-5'),

*CCND1* (forward 5'-TCTACACCGACAACTCCATCCG-3' and reverse 3'-TCTGGCATTTTGGAGAGGAAGTG-5').

### Cell cycle analysis

The HNSCC cells were seeded into 6-well microplates at a density of 5 × 10^5^ cells/ml. After 24 h, the cells were exposed to 5 and 15 μM of RIN-1 and incubated for 48 h. Next, the medium was removed, the cells were washed with PBS (without Ca^2+^ and Mg^2+^) and collected in a 5 mM solution of EDTA in PBS (without Ca^2+^ and Mg^2+^). After centrifugation (500 g, 5 min), the cells were fixed with 70% ice-cold ethanol and stored at − 20 °C. PI/RNase (PI/RNase Staining Buffer, BD Pharmingen, Catalogue Number 550825, Franklin Lakes, New Jersey, U.S.) staining was performed directly before the flow cytometric analysis (BD FACSCalibur, CellQuest Pro Version 6.0. software for the Macintosh operating system.). The PI fluorescence intensity of individual nuclei was determined and at least 10,000 events were measured within an acquisition rate of 100–300 events/s.

### Hoechst 33342 chromatin condensation staining

The HT-29 cells were plated onto cell culture dishes at a density of 2.5 × 10^5^ cells/ml. On the next day, the cells were exposed to a proper concentration of Notch modulators and were incubated for 48 h. Next, the medium was removed and the cells were washed with 0.5 ml of pre-warmed PBS (with Ca^2+^ and Mg^2+^) and incubated with the Hoechst 33342 staining solution (0.24 mg/mL of Hoechst 33342 in a serum-free culture medium) in darkness for 5 min. Chromatin condensation was assessed using confocal fluorescence microscopy (EVOS M5000 Image System; ThermoFisher). Bright blue fluorescing cells were recognized as apoptotic.

### Immunofluorescence staining (IFs)

For immunofluorescence (IF) staining of NOTCH1, E-cadherin, and F-actin, UT-SCC-42A cells were fixed with 3% paraformaldehyde and blocked with phosphate buffer saline (PBS) containing 3% bovine serum albumin (BSA). After blocking, cells were labeled with anti-NOTCH1 antibody (R&D Systems, Minneapolis, MN), anti-E-cadherin (R&D Systems, Minneapolis, MN), and anti-F-actin antibody (R&D Systems, Minneapolis, MN). Highly cross-adsorbed Alexa Fluor 633 donkey anti-goat IgG (H + L), Alexa Fluor 568 goat anti-rabbit IgG (H + L) and Alexa Fluor 488 donkey anti-sheep IgG (H + L) (Invitrogen, Carlsbad, CA) were used as secondary antibodies. The cells were mounted in Mowiol-DABCO (Sigma-Aldrich) and examined with Zeiss LSM510 META confocal microscope (Carl Zeiss, Jena, Germany).

For HES1 IF staining, HNSCC cell lines were seeded on glass-bottom Labtec 8-chamber slides (Nunc) at a density of 4 × 10^5^ cells/mL. After 24 h, the cell culture medium was removed and replaced by fresh medium (control) or medium containing 5 µM of RIN-1. After 48 h of incubation, the cells were washed twice by PBS buffer and fixed by 1:1 Acetone: Methanol fixation solution for 30 min at −20 °C. Cells were washed again in PBS buffer and incubated in Blocking Buffer (BB) for 1 h at room temperature. Immunofluorescence staining was performed by overnight (4 °C) incubation with primary anti-HES1 antibodies (Cell Signalling Technology, diluted 1:500 in BB) and, after washing three times in PBS, followed by incubation with Horseradish peroxidase-conjugated (HRP) secondary F(ab’)_2_ fragments of Anti-Rabbit IgG (1 h of incubation at room temperature, IgG dilution 1:1000 in BB). After triple PBS wash, to enhance the efficiency and signal strength of the reaction, cells were stained with Alexa Fluor 488 Tyramide Reagent (Invitrogen) following to manufacturer’s protocol. The reaction was stopped through incubation in 3% H_2_O_2_. For additional staining of nuclei, cells were washed in PBS with Hoechst 33342 at a concentration of 10 µg/mL. Next, slides were protected by being mounted with ProLong Gold mounting medium and visualized by a Nikon ECLIPSE Ti confocal microscope.

### Zebrafish xenograft experiments and ethics

The zebrafish strains used in the present study were obtained from the animal center at the Medical University of Lublin. Adult zebrafish (*Danio rerio*) were raised at 28.5 °C under 14 h of light and 10 h of the dark cycle. Embryos were maintained in the E3 buffer. The RIN-1 drug toxicity assay in zebrafish was performed according to Ali, Z. et al.^20^. Vybrant DiD (Invitrogen) stained UT-SCC-42B cells were cultured, implanted in the 2 days old zebrafish embryos, and observed and analyzed as previously described^21–23^. All experiments were approved by the Medical University of Lublin's Animal Research Ethics Committee.

### Western blotting

After sample preparation, protein lysates were obtained by lysing cells with RIPA Buffer (Thermo Scientific). Next protein level were quantitated by using Pierce Bicinchoninic Acid (BCA) PROTEIN Assay Kit (Thermo scientific) according to the manufacturer’s protocol. Based on the concentration measurements, equal amounts of protein were taken from each sample and prepared for standard SDS-PAGE polyacrylamide gel electrophoresis and western blotting, based on Bio-Rad Western Blotting Protocols (Bio-Rad). The membranes were imaged with a Li-COR Odyssey Infrared Imaging System to evaluate the protein expression. For western blotting, the primary antibodies used in this study were: HES5 (EPR15578) rabbit, NOTCH1/N1-TMICD (D1E11 XP(R)) rabbit mAb, NOTCH3/N3-TMICD (D11B8) rabbit mAb, HES1 (D6P2U) rabbit, JAG1 ((D4Y1R) XPCR) rabbit mAb. HES5 Ab was purchased from Abcam, (Cambrige, MA, USA), all other primary antibodies were purchased from Cell Signalling (Beverly, MA, USA). β-actin (C4) mouse monoclonal IgG1 (Santa Cruz Biotechnology) was used as house-keeping gene. The secondary antibodies used were anti-rabbit-IR800 (Li-Cor Biosciences, Lincoln, NE) and anti-mouse IgG (H + L) (DyLight 680 conjugate) (Cell Signalling Technology).

### Statistical analysis and preparation of graphs

Statistical analyses of all samples were performed using GraphPad Prism 8.0 (GraphPad Software Inc., California, U.S.A). One-way ANOVA followed by Tukey’s *posthoc* test and column statistics were used for comparisons (*p ≤ 0.05; **p ≤ 0.01; ***p ≤ 0.001 was considered statistically significant). All tests were performed in triplicates, at least. Biorender, Inkscape, and ImageJ-Fiji software were used to prepare the figures.

## Results

### Expression of key elements of Notch signalling in HNSCC cells

Firstly, we assessed the activity of the Notch signalling pathway in our representative panel of HNSCC cell lines. The qRT-PCR analysis showed that the tested HNSCC cells expressed different levels of mRNAs (Supplementary figure IA) and proteins (Supplementary figure IB) related to the Notch signalling pathway. Altogether, mRNA and protein expression data confirm that the Notch signalling pathway is fully active and functional in all 4 cell lines tested, and likely responsive to NOTCH modulators.

### Effects of modulators on the Notch signalling in 2D cultures

**Figure 2:**
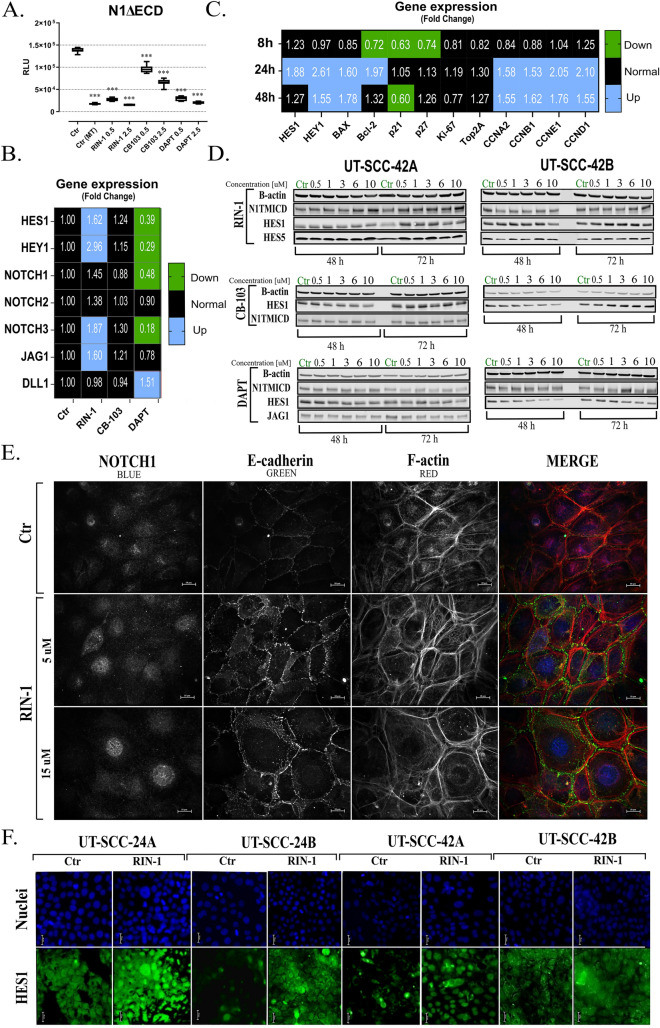
Modulation of Notch signalling by RIN-1, CB-103 and DAPT**,** in cells. HEK293 cells expressing 12xCSL-Luc reporter were transfected by the N1Δ-ECD and empty plasmid (mock transfection), and subsequently treated by Notch modulators. The relative luminescence units (RLU) level were measured in cells 48 h after activation by 0.5 and 2.5 µM of each Notch modulator (**A**). The effects of 2.5 µM of each of the Notch modulators on Notch signalling downstream genes are shown after 72-h of treatment (**B**) and time-course effect of 5 µM exposure of RIN-1 are shown (**C**) in UT-SCC-42B cells, as analysed by the qRT-PCR (2^−∆∆Ct^) method. Fold change value in the range of 0–0.749; was considered as downregulated (green, *p ≤ 0.05) and 1.501–10 as upregulated (blue, *p ≤ 0.05), respectively. The protein levels in UT-SCC-42A and UT-SCC-42B cells after 48-h and 72-h exposure to Notch modulators were also assessed by Western blotting (**D**). Equal loading amount of protein was verified by internal control (β-actin). N1-TMICD, (transmembrane and intracellular part of NOTCH1): cleaved form of NOTCH receptor. Impact of RIN-1 exposure on the intracellular expression and localization of NOTCH1 (blue), E-cadherin (green), and F-actin (red) in UT-SCC-42A cells (**E**). Target proteins were immunostained with fluorescently labelled antibodies and visualized in a confocal microscope. Immunofluorescence (IF) staining of HES1 (green) protein in HNSCC cell lines (**F**). After 48 h incubation with 1 µM RIN-1, cells were incubated with primary anti-HES1 and secondary HRP-conjugated antibodies. Signal was enhanced by cell staining with Alexa Fluor 488 Tyramide Reagent. For better visualization, DNA-dye Hoechst 33342 was used as a counterstain for nuclei (blue) staining. Representative photos of the HNSCC cell lines are shown. Ctr: control, HES1: anti-HES1, stained cells. Original blots are presented in Supplemental Figure VII.

To measure modulation of Notch signalling by compounds **RIN-1**, **CB-103**, and **DAPT,** we used HEK293 cells with stable expression of a Notch-responsive 12xCSL-Luc reporter system in which the activity of NICD/RBP-J/MAML correlates with the expression and activity of Firefly Luciferase. To intensify Notch signalling, HEK293 cells were transfected with a DAPT-sensitive, ligand independent, artificially constructed NICD1 fragment (N1ΔECD; Fig. 2A). As expected, cells transfected by N1ΔECD (Ctr) showed a clear increase in luciferase signal compared to mock-transfected cells (Ctr MT). Similarly, an expected decrease in the luciferase signal was observed after the use of reference compound **DAPT**, a well established γ-secretase inhibitor with consistent inhibitory effects on Notch signalling. Among the Notch modulators tested, a dose-dependent reduction in luciferase signal for both **CB-103** and **RIN-1** was also observed. Our results essentially confirm the results obtained in experiments with no N1ΔECD-transfected HEK293 cells (Supplementary figure II).

To further validate the authentic effects of **RIN-1**, and **CB-103** as specific modulators of Notch signalling in HNSCC, a series of qPCR experiments were performed, based on the expression levels of Notch-receptors themselves, and Notch-regulated or responsive genes, including *HES1*, *HEY1*, *NOTCH1*, *NOTCH2*, *NOTCH3*, *JAG1* and *DLL1* mRNA level (Fig. 2B). After 72-h incubation of UT-SCC-42B cells with 2.5 μM **DAPT**, a significant decrease in the expression of *HES1*, *HEY1*, *NOTCH1*, *NOTCH3* (Fig. 2B), and *HES2*, *HES4, HES5* and *HEY2* (Supplementary figure III) mRNA levels was observed, confirming its ability to inhibit Notch signalling. However, no significant changes in mRNA levels were observed for cells treated with **CB-103**. Interestingly, **RIN-1** showed a very prominent increase of mRNA levels for several NOTCH-responsive genes, especially for *HES1*, *HEY1*, *NOTCH3*, *JAG1* in the UT-SCC-42B cell line, pointing to its likely function as a NOTCH activator in this setting. Further analysis showed a time-dependent tendency of **RIN-1** to increase the expression of *HES1*, *HEY1*, and cyclins D1, E1, A2, and B1 (*CCND1*, *CCNE1*, *CCNA2*, and *CCNB1*) mRNA expression level (Fig. 2C) and *HES5*, *HEY2* and *HEYL* (Supplementary figure III).

To validate the results observed with compounds, protein expression analysis was performed (Fig. 2D). As expected, **DAPT** treatment decreased HES1 expression in HNSCC cells. Additionally, dose-dependent downregulation of HES1 was observed with a 48-h exposure to **CB-103**. However, this effect was not observed after 72-h exposure. In the case of **RIN-1**, the results revealed a tendency to increased N1-TMICD levels after 48 h and 72 h incubation in UT-SCC-42A cells and after 72 h incubation in UT-SCC-42B cells. Additionally, increased HES1 expression was observed after 72 h incubation in both HNSCC cell lines. Taken together, our data indicate that **CB-103** be considered as a functional inhibitor and **RIN-1** as a specific activator of Notch signalling in 2D cultures of HNSCC cells.

The compound **RIN-1** also showed a significant increase or induction of the Notch signalling pathway at both the mRNA and protein levels. Simultaneously, **RIN-1** reduced the signal in the NOTCH-specific luciferase reporter assay. To explain this phenomenon, we investigated the effects of **RIN-1** on the subcellular localization NOTCH localization. For this purpose, immunofluorescence staining (IFs) of NOTCH protein, and the cytoskeleton (E-cadherin, and F-actin) was performed (Fig. 2E). UT-SCC-42A cells treated with **RIN-1** showed a differential localization of NOTCH protein and numerous morphologic changes, compared to untreated cells. The cells become larger and more regular (honeycomb shape), possibly indicating a higher level of squamous differentiation. Most importantly, the number of NOTCH1-positive stained cell increased significantly, indicating the accumulation of transcriptionally active NICD1 in the nuclei. Additionally, increased expression of E-cadherin was observed, which was co-localized with F-actin near the cellular membrane, indicating a cortical actin cytoskeleton.

To confirm the potential of **RIN-1** to specifically activate Notch signalling, also HES1 expression level was tested. As expected, and based on our previous data, HNSCC cells showed increased expression of HES1 protein with a dominant nuclear localization, increased in cells treated with 1 µM **RIN-1** compared to untreated cells (Fig. 2F).

### Effect of Notch-targeting drugs on the growth of HNSCC cells in 2D cultures

**Figure 3:**
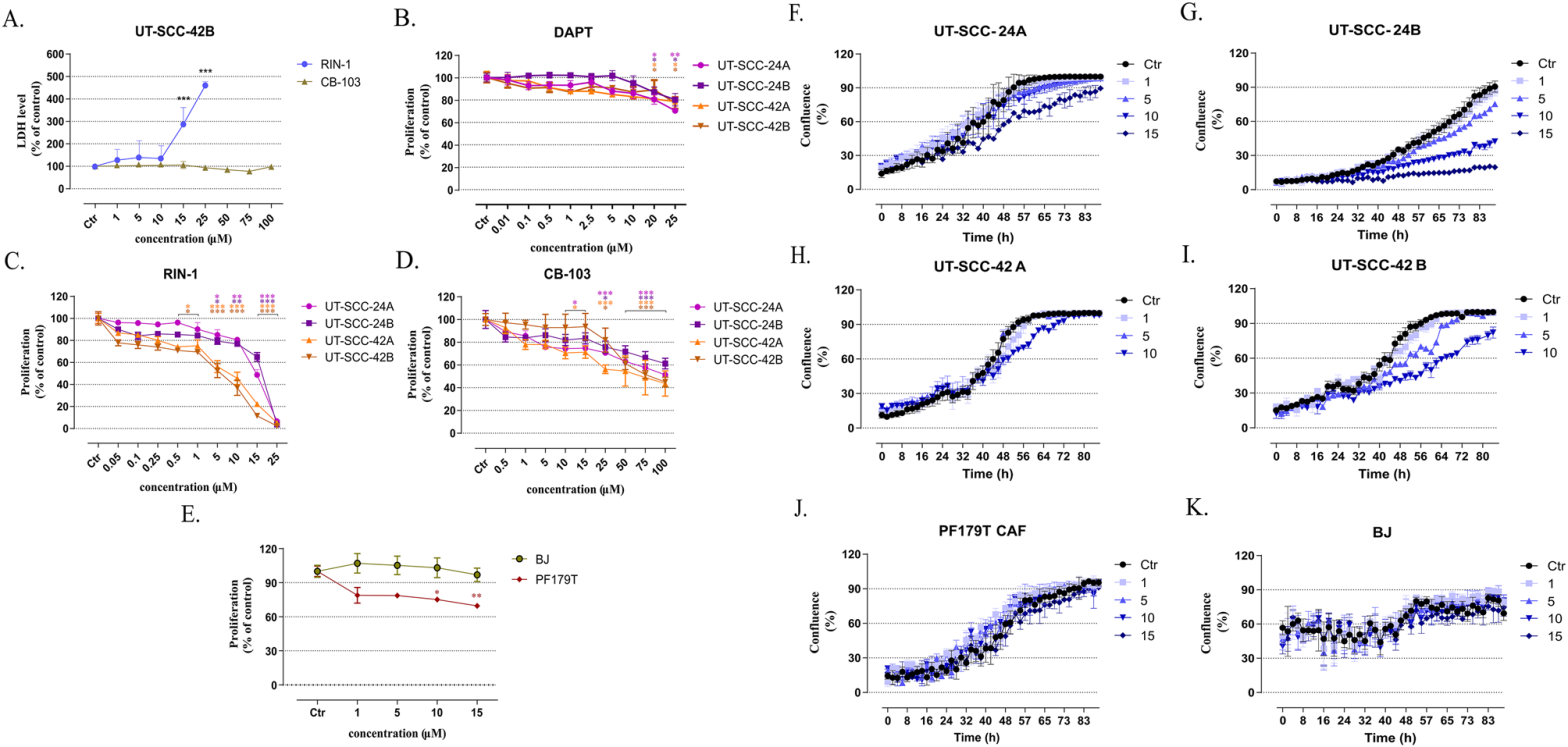
The effects of Notch signalling pathway modulators on cell viability, growth and proliferation of HNSCC, compared to non-transformed, cancer-associated fibroblasts (PF179T CAF), and normal foreskin fibroblasts (BJ) in 2D cultures. The influence of Notch signalling modulators on the permeability of cell membrane (**A**). UT-SCC-42B cells were incubated with RIN-1 (1–25 µM), and CB-103 (15–100 µM), respectively. The relative proliferation of HNSCC cell lines assessed by using the MTT assay after 96-h of drug exposure (**B**–**D**). The results represent the mean growth reduction ± SD (% of control). The number of viable PF179T and BJ cells was assessed with the WST-8 assay after 72-h drug exposure (**E**). The proliferation of RIN-1-treated cells examined using the Incucyte real-time imaging system (**F**–**K**). The percentage of confluency is shown.

To evaluate the impact of compounds **RIN-1**, and **CB-103** on cancer cell membrane permeability (and thus cell viability), the LDH release assay was performed (Fig. 3A). Compared to control, a statistically significant increase in LDH release was observed for comparatively high concentrations between 15 and 25 µM of **RIN-1**, after 48 h hours of drug exposure. No significant changes in LDH release were observed after **CB-103** treatment. Significant increase in LDH release was also observed for **RIN-1** (15–25 µM), after 24 h hours of drug exposure (Supplementary figure IV A). We conclude that none of the drugs had any strong, non-specific toxicity and none showed an impact on membrane integrity or permeability at concentrations below 10 µM.

Next, the chemosensitivity of HNSCC cells responding to Notch signalling pathway modulators was evaluated by using the MTT assay (Fig. 3B–D). For each compound tested, a dose-dependent decrease in the number of viable cells was observed after a 96-h drug exposure. The highest activity was observed for UT-SCC-42A and UT-SCC-42B cell lines exposed to **RIN-1**, for which the IC_50_ value was estimated to be 3.64 and 2.05 µM, respectively (Supplementary Table S1). Interestingly, in the WST-8 test, no reduction in the number of viable BJ cells, and only a slight, but statistically significant decrease in the number of viable PF179T cancer-associated fibroblast (CAF) cells was observed for **RIN-1** at the concentration range between 1 and 15 µM (Fig. 3E). This indicates potential cancer-specific effects of the compounds (confirmed also in WST-8 assay; Supplementary figure IV B) that are not shared by non-transformed, non-cancer cells and cell lines.

Additionally, the effect of **RIN-1** on HNSCC, cancer-associated fibroblasts (PF179T CAF), and the proliferation of normal fibroblasts (BJ) were investigated in 2D cultures, by monitoring cell growth until reaching confluency. As expected, a significantly decreased HNSCC cell proliferation (% of cell confluence) was observed after **RIN-1** treatment (Fig. 3F–I). Additionally, both metastatic UT-SCC-24B (Fig. 3G) and UT-SCC-42B (Fig. 3I) cell lines showed higher sensitivity to **RIN-1**, compared to primary UT-SCC-24A (Fig. 3F) and UT-SCC-42A (Fig. 3H), respectively. Compared to untreated control cells, PF179T CAF and BJ cells showed no significant changes in cell proliferation after **RIN-1** treatment (Fig. 3J–K).

### Influence of Notch-targeting drugs on HNSCC cells migration, cell cycle and apoptosis

**Figure 4:**
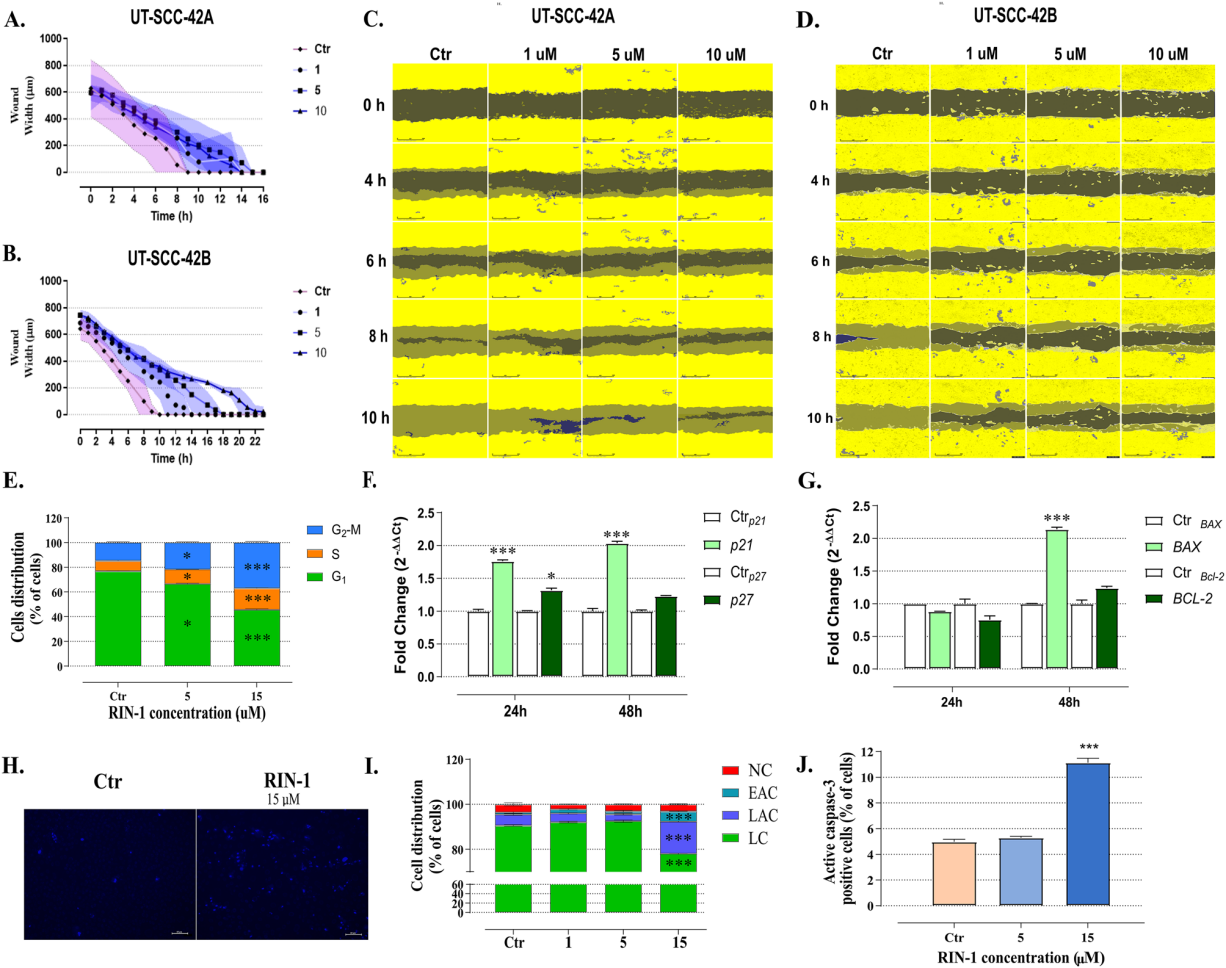
Influence of RIN-1 on HNSCC cells migration, cell cycle and apoptosis. A graphical representation of the reduction in wound width (μm) over time during the treatment with Notch modulators is shown (**A**–**B**). Representative wound closure images show UT-SCC-42A (**C**) and UT-SCC-42B (**D**) cells treated with RIN-1 over different incubation times (full confluence: yellow, wound: dark area, wound closure: yellow- gray area). Effects of RIN-1 treatment on UT-SCC-42B cell cycle (**E**). The percentage of cells in each phase of the cell cycle in FACS analysis is marked by the propidium iodide (PI) staining method. Cells were treated with 5 and 15 μM of RIN-1 for 48 h. The effect of 15 µM of RIN-1 exposure (24 and 48 h) on mRNA expression of *p21 (CDKN1A)* and *p27* (*CDKN1B*) (**F**) and *BAX* and *BCL-2* (**G**), of UT-SCC-42B cells was assessed by the qPCR method. Representative pictures of UT-SCC-42B cells stained with Hoechst 33342 dye after 48 h exposure to 15 µM RIN-1 are shown (**H**). Chromatin condensation is shown as intense bright-blue fluorescence. The percentage of late apoptotic (LAC; Annexin V^+^/PI^+^), early apoptotic (EAC; Annexin V^+^/PI^−^), necrotic (NC; Annexin V^−^/PI^+^), and viable (LC; Annexin V^−^/PI^−^) UT-SCC-42B cells were assessed by Annexin V and Propidium Iodide (PI) staining assay and analysed with flow cytometry (**I**). Activation of caspase-3 in UT-SCC-42B cells is shown after 48 h of RIN-1 treatment (**J**).

To assess changes to the invasive or migration potential of HNSCC cells exposed to Notch signalling modulators, “scratch wound” or “wound healing” assays were performed (Fig. 4A–D). The non-treated, rapidly migrating UT-SCC-42A and UT-SCC-42B cells closed the artificial wound area already 8–10 h after seeding. Untreated UT-SCC-42A closed the wound area 14–16 h after wound formation. A pronounced, dose-dependent effect of **RIN-1** was observed (Fig. 4A, C). UT-SCC-42B cells treated with 1, 5, and 10 µM **RIN-1** effectively closed the wound area 14, 17, and 22 h after wound formation, respectively (Fig. 4B, D), compared to 8–10 h for untreated cells. Changes in wound closing kinetics were not observed for any HNSCC cells treated with **CB-103** (Supplementary figure V A). We conclude that cell motility or migration was specifically targeted only by **RIN-1**, but none of the other compounds. This effect was most prominent in the UT-SCC-42B cells, which are highly aggressive and of metastatic origin.

The ability to induce programmed cell death or apoptosis of cancer cells is one of the most common mechanisms of action of anti-cancer drugs accompanied by a disturbance in cell cycle, growth, and decrease in the number of cancer cells. Therefore, we assessed the response of UT-SCC-42B cells exposed to intermediate (5 µM) to very high concentrations (> 10 µM) of **RIN-1** in all phases of the cell cycle. We deliberately exceeded for physiologically active drug concentrations to test for the onset of overt cytotoxic responses. 48-h exposure of 5 and 15 μM **RIN-1** to UT-SCC-42B cells showed a dose-dependent accumulation of cells in the S and G_2_-M phases of the cell cycle, with a concurrent decrease in the number of cells in G_1_ phase. The most visible changes were observed at a comparably high concentration of 15 μM, at which the number of cells in S and G_2_-M increased from 8.30 ± 0.33% and 14.72 ± 0.56% in the control cells to 17.56 ± 0,42% and 36.93 ± 0.88% respectively, in treated cells. Simultaneously, the number of cells in the G_1_ phase decreased from 76.97 ± 0.25% in the control, to 45.49 ± 0.75% in treated cells (Fig. 4E). To represent the molecular mechanism of **RIN-1** action on UT-SCC-42B cell cycle, the gene expression analysis was carried out using the qPCR method. Compared to untreated UT-SCC-42B cells, 15 µM of **RIN-1** also caused a significant increase in p21 (*CDKN1A*) expression after 24- and 48-h incubation (Fig. 4F). Additionally, 48-h of incubation of UT-SCC-42B cells with 15 µM **RIN-1** showed increased *BAX* (*BAX;* apoptosis-related BCL2 associated X, apoptosis regulator) levels with a *BAX* to *BCL-2* mRNA ratio equal to 1.73 (Fig. 4G).

One of the key features of apoptosis is intense chromatin condensation and formation of apoptotic bodies, which can be detected by Hoechst 33342 DNA staining. To evaluate the pro-apoptotic features of Notch signalling modulators, UT-SCC-42B cells were incubated with rising concentrations of 1 to 15 µM of **RIN-1**. Compared to untreated control cells, no significant condensation of chromatin was observed at the concentration range between 1 and 10 µM (Supplementary figure V B). Intense chromatin condensation was observed in UT-SCC-42B cells after treatment with 15 µM of **RIN-1** (Fig. 4H), which can be interpreted as a sign of induction of apoptosis.

**RIN-1** pro-apoptotic properties were investigated in Annexin V and Propidium Iodide (PI) staining assay. Strong induction of the apoptosis rate (total level of early and late apoptosis cells) from 5.78 ± 0.63% in the control to 18.9 ± 0.21% in treated cells was observed for the UT-SCC-42B cell line after exposure to 15 μM of **RIN-1** (Fig. 4I). However, no increase of apoptotic cells was observed at lower and probably more physiological concentrations of **RIN-1** (< 10 µM). Similarly, no increase of apoptotic cells was observed for UT-SCC-24A, UT-SCC-24B and UT-SCC-42A cell lines (Supplementary figure V C-F).

Finally, to confirm that **RIN-1** induced apoptosis in UT-SCC-42B cells, the level of active caspase-3 was assessed. The 48-h exposure of **RIN-1** to UT-SCC-42B cells resulted in a statistically significant increase in the number of cells positive for active caspase-3, increasing from 4.98% in untreated control cells to 11.14% in cells treated with 15 μM **RIN-1** (Fig. 4J). No procaspase-3 activation was observed at lower **RIN-1** concentrations (1–10 µM), pointing again to the low non-specific cytotoxicity of the compound.

### The effect of Notch signalling pathway modulators on growth of HNSCC spheroids formed in non-adherent conditions and HNSCC organoids, formed in matrix-embedded 3D cultures

Despite their advantages, experiments conducted in a 2D monolayer culture, adhered to plastic support carry several disadvantages related to, among others, the strong dependence on cell adhesion-mediated signalling of growing cells. Due to the high affinity of Notch signalling to physical stimuli in 2D environment, we also evaluated the activity of Notch modulators in low- or non-adherent conditions, resulting in the formation of 3D spheroids that result from spontaneous aggregation of tumour cells. These aggregates are functionally very different from the organoids formed after embedding single tumour cells in laminin-rich matrix like Matrigel (see below).

**Figure 5:**
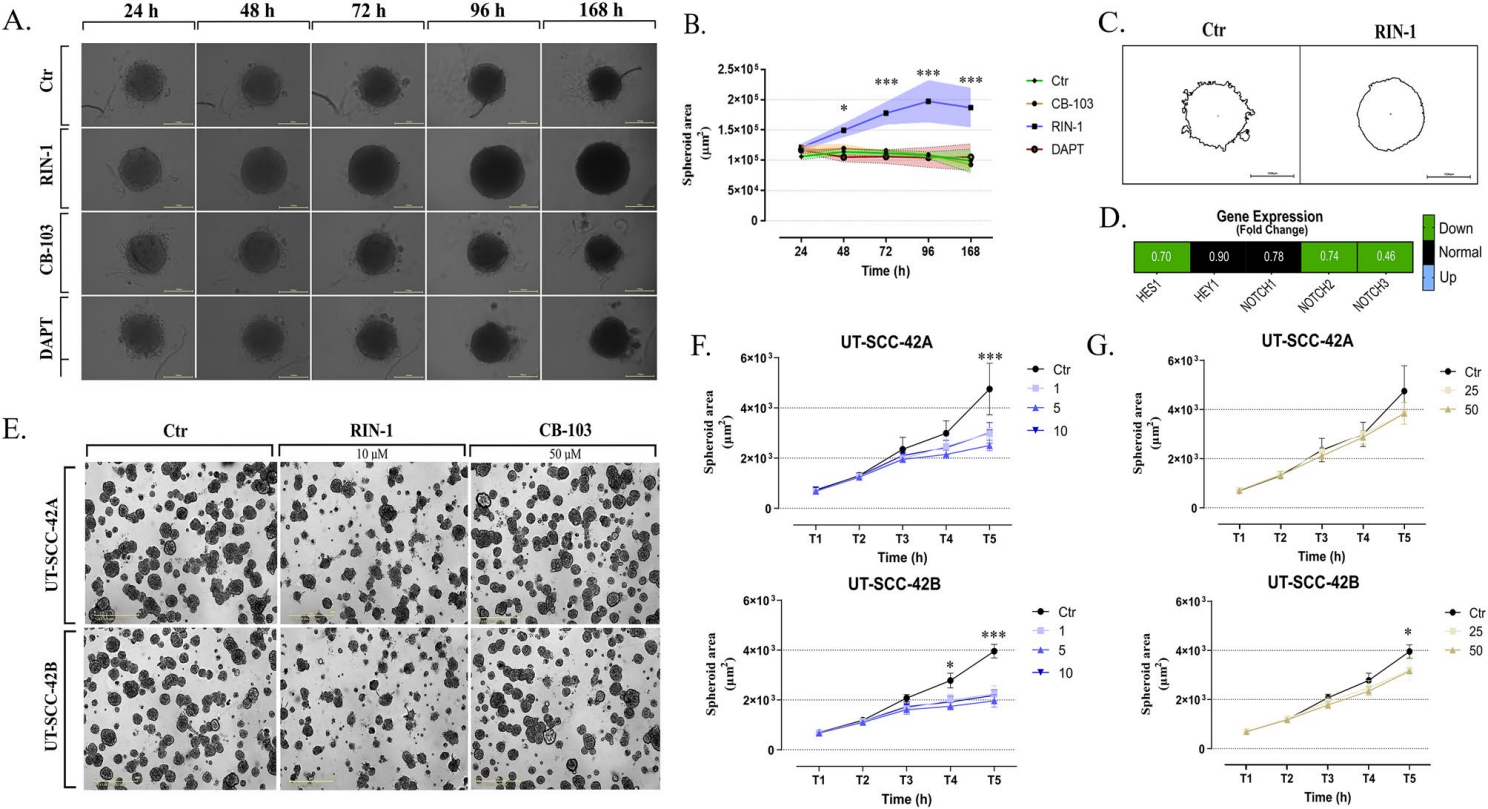
The effect of Notch signalling pathway modulators on growth of HNSCC spheroids and organoids. After suspending in non-adherent growth conditions, UT-SCC-42B cells were left to aggregate with each other, simultaneously treated with Notch modulators and observed for 168 h. Representative photography of UT-SCC-42B re-aggregated spheroids after exposure to 2.5 µM of Notch modulators (**A**). Graphs of the average size of drug-exposed UT-SCC-42B spheroids (mean ± SD; μm^2^) over time (**B**). Comparison of the shape of single spheroids treated with 2.5 µM of RIN-1, compared to the untreated control (**C**). Effect of 2.5 µM of RIN-1 treatment on Notch signalling and mRNA expression of downstream genes after 72-h marked by qPCR (FC; 2^−∆∆Ct^ method) in non-adherent conditions (**D**). Fold change (FC) for each gene indicated in relation to RIN-1 untreated control (set to 1) in UT-SCC-42B cells. Impact of Notch modulators on growth, proliferation and morphogenesis of mature organoids in 3D cultures (**E**–**G**). After seeding single cells into the three-dimensional (3D) sandwich model, HNSCC organoids were allowed to grow and mature for 3 days. Next, organoids were treated (time point T2) with Notch modulators. The endpoint of each experiment was established three days after drug treatment (144 h after seeded cells; T5). Representative images show untreated and drug exposed HNSCC organoids at the endpoints of experiments (**E**). Graphs indicate the average size of organoids (mean ± SD; μm^2^) over time for RIN-1 (**F**), and CB-103 (**G**).

After 24-, 48-, 72-, 96- and 168- hours of incubation of spheroids formed by UT-SCC-42B cells with 2.5 µM each of **CB-103** and **DAPT**, no significant changes in size were observed. In contrast, significant induction of the growth of spheroids was observed after exposing the cells to 2.5 µM **RIN-1** (Fig. 5A,B). Additional morphometric analysis showed more uniform spheroid shape, compared to the untreated control (Fig. 5C). More uniform spheroid shape, compared to the untreated control, was also observed for CAFs after **RIN-1** treatment, however, no significant effect of Notch modulators on CAFs spheroids growth was observed (Supplementary figure VI A,B). Finally, in relation to the high activity of **RIN-1** in UT-SCC-42B spheroids, the level of Notch signalling components was assessed by qPCR. Interestingly, under these special conditions, **RIN-1** treatment resulted in down-regulation of both *HES1*, *NOTCH2* and *NOTCH3* mRNA level compared to the untreated control cells (Fig. 5D), indicating inhibition of Notch signalling in UT-SCC-42B cells spheroids. This is striking contrast to our observations in 2D monolayer cultures. **RIN-1** may thus act as a bimodal modulator that can affect NOTCH activity and expression of NOTCH-responsive genes in both directions, depending on the cellular differentiation state, cell culture conditions and the baseline levels of NOTCH pathway activities in each of these conditions.

To evaluate the effect of treating HNSCC cells with Notch modulators in physiologically relevant growth conditions that are more closely related to solid tumours, a series of experiments using three-dimensional (3D) cultures of organoids, embedded in laminin-rich matrix such as Matrigel, were prepared. Using the 3D sandwich assay developed by our laboratory, most organoids formed in 3D cultures resulted from single tumour cells embedded into Matrigel. Most of these organoids showed significant epithelial maturation and polarization, as reported elsewhere^24–26^ indicating the strong differentiation-promoting effect of laminin-rich ECM like Matrigel.

To indicate the effect of Notch modulators on mature, well-differentiated and polarized tumour organoid structures, 3-day-old HNSCC organoids were subjected to the **RIN-1** and **CB-103** treatments. Subsequent changes to organoid morphology and growth were monitored for up to 3 days, again using the Incucyte system. Under these conditions, a decrease in the total area of organoids (growth; Fig. 5E–G) was observed. The most prominent drug activity was observed for **RIN-1** (Fig. 5E, F). Relatively low or absent activity was again characteristic of **CB-103** (Fig. 5G). In these experiments, no significant differences were observed between primary and metastatic/recurrent HNSCC cell lines (Supplementary figure VI C). Dose- and time-dependent inhibition of organoid growth was observed for the compound **RIN-1,** also when added early (at day 1, simultaneous to seeding; Supplementary figure VI D-F) to cell cultures.

### RIN-1 antitumour activity in the Danio rerio zebrafish model

**Figure 6:**
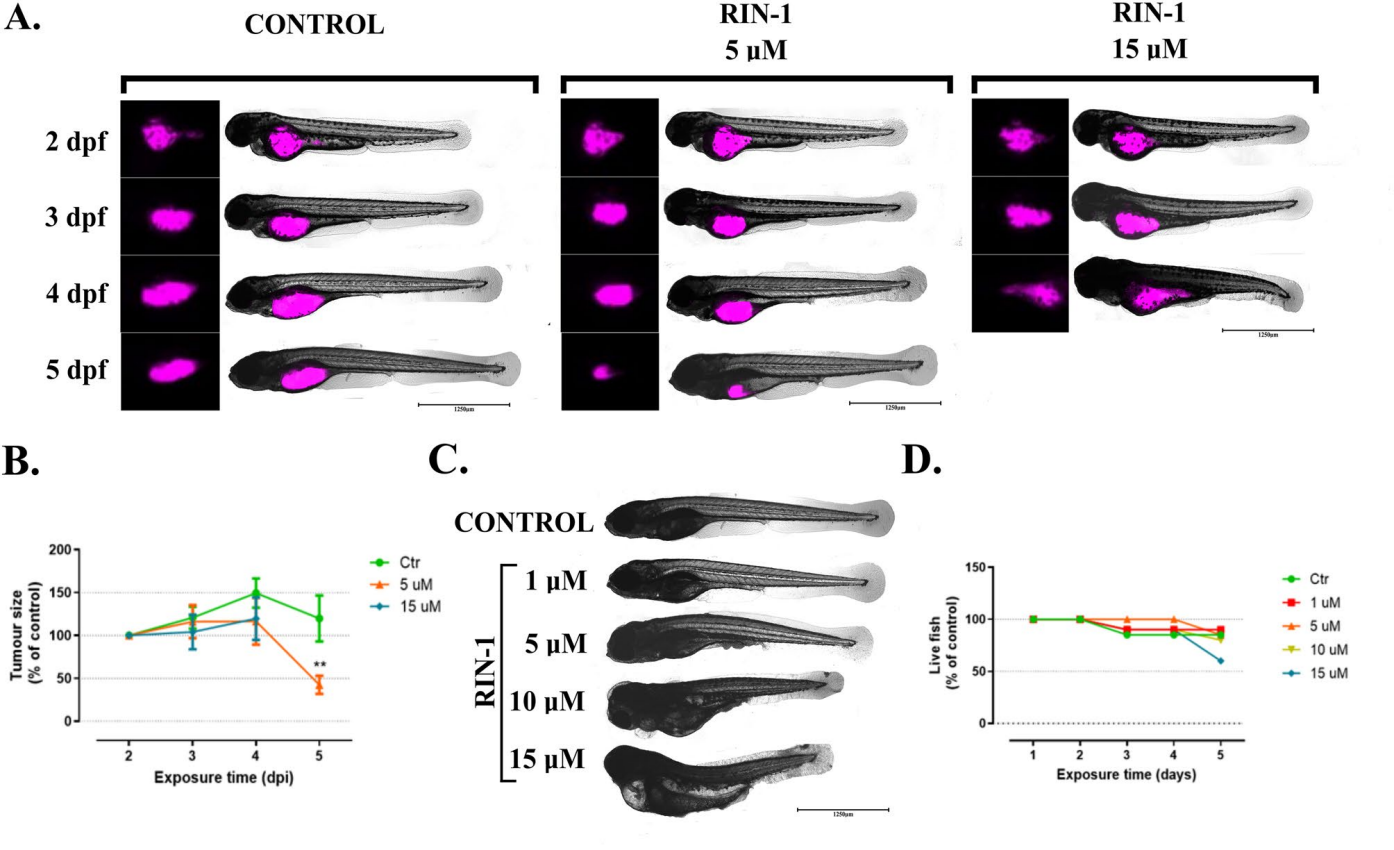
RIN-1 in vivo activity in the *Danio rerio* model. The effects of RIN-1 treatment on UT-SCC-42B cells xenografts (**A**, **B**). After Vybrant™ DiD-staining, 2500 UT-SCC-42B cells were injected in 2-days old (2 dpf; days postfertilization) fish larvae, and incubated with 5 and 15 µM of RIN-1 for 3 days. (**A**) Representative pictures taken 2–5 days postinjection (dpi) UT-SCC-42B cells into larvae. Tumour size (total xenografts area; n ≥ 3 fish) 2, 3, 4, and 5 dpi, based on quantification of Vybrant DiD fluorescence, shown as % of control (xenografts size at time 2 dpi calculated as 100%) (**B**). Effect of RIN-1 on the surviving larvae (**C**, **D**). Representative 5-day-old photos of larvae taken after 3-day treatment with RIN-1 (**C**). Graph showing the viability of *Danio rerio* larvae (**D**). Two-day-old larvae were treated with 1, 5, 10, and 15 µM RIN-1, respectively, and observed for 3 days (n = 20 larvae per group).

The *Danio rerio* zebrafish model was used to investigate the anti-tumour potential of **RIN-1** in in vivo conditions. **RIN-1** in E3 buffer was added to the 2-days old (2 dpf) fish larvae immediately after UT-SCC-42B cells injection. Next, the fish were monitored for 3 days every 24 h. An increase in tumour size over time was observed, in both the control and **RIN-1** treated fish, during the first 48 h after HNSCC cells injection. The most significant tumour growth was observed in the control group after 48-h of growth, in which the tumour size increased by 49.6 ± 17.04% (comparing time-points 2 vs 4, Fig. 6A, B). In the same time frame, a significantly reduced growth of 16.5 ± 27.28% and 19.7 ± 24.79% was observed in fish treated with 5 and 15 μM **RIN-1**, respectively. **RIN-1** exposure therefore resulted in significantly reduced tumour growth. After 72 h (5 dpf) of incubation with 5 μM of **RIN-1**, a reduction to 42.49 ± 10.64% of the original tumour size was observed (Fig. 6B). The tumour size also decreased in the control group after 72 h of incubation, but to a smaller degree (20 ± 26.98%). Prolonged incubation (72 h; 5 dpf) with 15 µM **RIN-1** led to serious morphological changes and toxicity to the fish which made it impossible to determine the tumour size.

In order to investigate the influence of **RIN-1** on the viability and morphology of the *Danio rerio* larvae, we performed additional microscopic observations of in vivo tumour formation (Fig. 6C, D). For these purposes, **RIN-1** was applied to healthy, non-tumour-bearing 2-day-old larvae. Next, fish were observed for 3 days. At a concentration of 1–5 µM, no severe changes in the morphology of the larvae were observed. However, concentrations of 10 and 15 µM **RIN-1** resulted in severe changes in the development of the larvae such as a curved and shortened body, yolk sac edema, and pericardial edema (Fig. 6C). **RIN-1** did not reduce the viability of fish at concentrations between 1 to 10 µM (observation based on the work of the circulatory system), but increased toxicity was regularly observed after 72 h exposure to 15 µM (Fig. 6D).

## Discussion

Scientific evidence suggests that in HNSCC, Notch signalling may have a bimodal, dual function as either a tumour suppressor or as a protooncogene^6,7,9,10^, at different stages of tumour progression and development. The tumour suppressor function of Notch signalling is confirmed by a multitude of observations that central players of the Notch pathway regulation are frequently inactivated by loss-of-function (LoF) mutations, preferentially at early stages of tumour initiation and development. *NOTCH1* is the second most frequently mutated gene in HNSCC^6^, after TP53; resulting in frequent aberrations in intracellular signalling. The LoF are often homogeneous, or supported by simultaneous epeigenetic silencing of the second NOTCH1 allele. The lack of functional NOTCH1 in a large fraction of HNSCC is observed at a range between 10–18%^1^ and 40%^2^, and the vast majority of these mutations are oncogenic LoF mutations^27^. Fifteen additional, potential tumour suppressor genes under the control of Notch or modulating Notch downstream functions have been identified in an animal model of HNSCC, including *Adam10*, *Ripk4, EP300*, and *Ajuba*^28^, which adds emphasis to the tumour suppressor function of Notch signalling. This phenomenon is observed across most squamous cell carcinomas also at other locations (e.g., ski, lung, uterine cervix, and esophagus), and may represent a hallmark of “squamousness”. Functional inactivation of Notch signalling, and specifically of the NOTCH1 receptor as a critical tumour suppressor, is characteristic for early-stage cancer development, targeting keratinocytes of the skin, lung, uterine cervix, esophagus, and the head and neck area.

On the other hand, subgroups of HNSCC patients with activating, or gain-of-function *NOTCH1* mutations have been observed as well, and these may show worse prognosis than tumours with wild-type *NOTCH1*^6^. Additionally, genes functional in regulating Notch signalling are often elevated in HNSCC compared to normal tissues, and inhibition of the Notch pathway was shown to reduce HNSCC cell growth and invasion^6^, again indicating an oncogenic potential for the Notch pathway in advanced HNSCC. This may be further supported by increased activity of other NOTCH receptors (NOTCH2, 3, and 4), although this is only poorly investigated in HNSCC.

It is currently unclear which percentage of HNSCC cases may retain functional Notch signalling, which receptor/ligand combinations are contributing to this activity. It is also unknown which genetic and epigenetic alterations in addition to NOTCH receptor mutations may further contribute to modulate Notch signalling activity, e.g., according to cues from the tumour microenvironment, tissue-specific differentiation, or a broad spectrum of stress response conditions, including hypoxia or response to chemotherapies. An integrated, functionally increased “generalized” Notch pathway activity may be highly relevant for the chemo- and radiosensitivity of cancer cells, for acquired drug resistance and tumour progression, relapse local and distant metastasis, or invasiveness. Relatively little is known about the continued, wild-type activity of NOTCH functions which are observed in a considerable fraction of HNSCC, even in the presence of frequent *NOTCH1* LoF mutations, and the potential mutual compensation or cooperation of the four NOTCH receptors 1–4 in cancer cells. It is currently unclear if there is a significant functional overlap or compensation between the 4 receptors, and the 5 ligands which are all widely expressed in both tumour and stromal cells. Last not least, there are currently no systematic, functional studies investigating the totality of Notch pathway activities across all the receptors, ligands, and various cell types involved in HNSCC. There is also a lack of reliable methods that would allow to quantitatively measure and quantify an overall, integrated Notch pathway activity in cancer cells and tissues.

Similarly, it remains unclear how tumour cells may react to increasing or decreasing NOTCH signalling activity, and the “gene dosage” effects of the Notch pathway in cells of squamous epithelial origin (= keratinocytes). The ambiguous and poorly understood, ambivalent role of Notch signalling in cancer initiation, development and progression severely limits its use as a molecular target for HNSCC treatment. Accordingly, there are no clinical trials testing Notch-pathway inhibitors in HNSCC currently ongoing. The most commonly used inhibitors are the γ-secretase inhibitors (GSI) such as DAPT, frequently used as a Notch pan-inhibitor^29^. However, γ-secretase is involved in processing signals from a series of other receptors^29^, thus affecting many different signal transduction pathways. The effects observed with GSIs may be only partially due to inhibition of Notch signalling. GSIs also have shown poor cancer and pathway specificity, they potentially impair the antitumour immune response and show a pronounced and debilitating gastrointestinal cytotoxicity^30^ that has precluded any successful clinical trials to date. For this and other reasons, no GSI have yet been approved for cancer therapies. The focus of anti-Notch drug discovery has therefore shifted to other mechanisms of action which may be interfered with, to either functionally block, inhibit or modulate Notch pathway activity. In this study, we have selected 2 of these “next generation” anti-Notch compounds, which may both show lower levels of systemic and non-specific toxicity, and a wider therapeutic window. In addition, blocking Notch signalling has been deemed counterintuitive for the therapy of HNSCC, since a considerable number of tumours harbor LoF mutations in Notch receptors or downstream regulators. Nevertheless, a considerable fraction of HNSCC retain functional or wildtype Notch pathway activities, while yet others may even show hyperactivation; and all 4 receptors may be involved or jointly effective in this effort. For these reasons, we have explored the potential of small molecule Notch modulators (including both inhibitors and activators) to interfere with cancer proliferation and progression in HNSCC, focusing primarily on tumours that lack any mutations in Notch pathway genes, and show continued and functional, intermediate to high-expression levels of NOTCH receptors. Accordingly, we selected HNSCC-derived cell lines (UT-SCC-42A, -42B, -24A and -24B) that show intermediate level, but functional Notch signalling, as representatives for a probably large fraction of HNSCC cases that do not show inactivation of the pathway, nor overt hyperactivation, as observed in TNBC. We pursued primarily the key question if interfering with active Notch signalling in HNSCC cell lines either way may have an impact on differentiation, growth and proliferation, apoptosis and cell cycle progression, and the tumour cell plasticity in general. Our central hypothesis was that Notch signalling may be particularly relevant for head and neck tumour cells that typically remain competent to engage in at least (partial) epithelial or squamous, show cell-type specific differentiation, or pronounced tumour cell invasion and motility culminating in distant metastasis. All of these phenomena are considered indications for increased tumour cell plasticity. Accordingly, we selected a broad range of differential cell culture conditions, including rapidly proliferating 2D monolayer cultures, non-adherent spheroid cultures (which typically result in de-differentiation), and organotypic 3D cultures, resulting in organoids embedded in differentiation -promoting extracellular matrix preparations such as Matrigel.

Among a larger collection of “next generation” drugs available for research purposes (including also SAHM1, FLI-06 and Yhhu-3792), we focused on a potential Notch signalling activator, **RIN-1**, and putative inhibitor, **CB-103** which were tested against HNSCC cells. We also used one GSI (DAPT) as a “conventional” anti-Notch drug, for comparison. Our study shows that modulation of Notch signalling by small molecule drugs can have significant anti-proliferative effects, thus limiting the growth of HNSCC cells both in 2D and 3D conditions. The effects of non-toxic concentrations of Notch signalling modulators (**RIN-1** < 10 µM) resulted generally in a reduction in the growth of HNSCC cells under both 2D and 3D conditions, almost regardless of the inhibitory or stimulatory nature of the compounds. This is pointing to the possibility that the 4 cell lines used in this study may have an “optimized”, physiologically ideal Notch pathway activity or gene dosage that provides them with growth stimulation and supports proliferation versus differentiation. Any deviation from these levels may result in reduced proliferation, increased squamous differentiation, and even cytotoxicity.

While a generalized antitumour effect of almost all Notch modulators seems to be relatively well documented in this manuscript, our understanding of the mechanism of action for the remaining compounds tested seems less clear. Despite a number of studies that confirmed the anti-tumour efficacy of **CB-103** in other types of cancer^13,31^, our research suggests an almost absent or very weak activity against HNSCC. This may be related to the fact that inhibition of Notch signalling by **CB-103** may be more effective in types of cancer such as T cell acute lymphoblastic leukemia (T-ALL), in which Notch signalling is clearly activated by GoF oncogenic mutations, resulting in hyper-activation of the Notch pathway. Comparable oncogenic activity or even dependency may be missing in HNSCC.

Of all the drugs tested, **RIN-1** showed the highest spectrum of specific activities and the most prominent and reproducible morphologic/phenotypic effects. Our analysis showed that **RIN-1** significantly activates Notch signalling in cultured HNSCC cells. This is also indicated by increased expression of Notch-dependent genes and proteins (HES1, HES5). Thus, it is surprising that the luciferase promoter signal was decreased in **RIN-1**-treated 12xCSL-Luc reporter cells, which contradicts an assumed activation of the NICD/RBP-J/MAML complex; although this may be partly dependent on the cell type used in the assay. The same reporter construct, transfected into keratinocytes or squamous epithelial cancer cells, may have resulted in different responses. Hurtado et al. suggest that **RIN-1** may induce changes in gene expression that resemble the silencing of RBP-J by siRNA^12^. Due to the fact that RBP-J is essential for the function of the NICD/RBP-J/MAML transcriptional regulator complex and the expression of NICD-dependent target genes, it is unexpected to observe lack of expression of luciferase signal after SHARP inhibition (RBP-J inhibitor) by RIN-1. Nevertheless, an increase in Notch signalling upon treatment with **RIN-1** cells was also observed in the present study. Thus, the presented results essentially support Hurtado et. al. observation, although our data suggest that **RIN-1** acts by new, not yet described molecular mechanism of action, e.g. like a SHARP inhibitor, consequently increasing the availability of free RBP-J for NICD.

At the same time, **RIN-1** reduces the level of migration and mobility of HNSCC cells in 2D culture model. We hypothesize that these effects are at least partially due to non-physiological high levels of Notch pathway activity in cancer cells, which may lead to “conflicting signalling”—simultaneously promoting cell proliferation and epithelial differentiation, or maturation. This is particularly pronounced in 2D conditions, where cells are strongly stimulated by the adherent growth on plastic surfaces. It is also possible that **RIN-1** affects the characteristic “tumour cell plasticity”, often observed in advanced cancer cells and cell lines, and which is generally promoting processes such as invasion and motility.

According to our data, anti-proliferative effects specifically by **RIN-1** treatment may result from both activation and inhibition of Notch signalling, dependent on cell culture conditions and microenvironment, but also the lineage-specific origin of the cells (e.g., squamousness). This potentially indicates that Notch signalling activity in HNSCC cells and cell lines is tightly controlled, and any interference—in either direction—may result in disturbing of cell growth and balance between proliferation and squamous differentiation. Particularly noticeable in this regard are the results obtained in non-adherent culture conditions, where a significant increase in the size and roundness of poorly differentiated tumour spheroids was observed that coincides with a putative induction or activation of Notch signalling by **RIN-1**. We hypothesize that this may result from disturbing the balanced homeostasis of Notch signalling, likely in connection with a number of other cell signalling pathways and control mechanisms. This may also include changes to cell metabolism (not investigated here), cell cycle progression, and (at higher concentrations of Notch modulators), also cell death and apoptosis.

A significant effect of **RIN-1** on the reduction of the tumour size was confirmed in the experiments with the *Danio rerio* zebrafish model. Additionally, at the concentrations where **RIN-1** was effective in reducing the size of the tumour, there was no significant reduction in the viability of the *Danio rerio* larvae. It should be kept in mind that Notch signalling plays a key role especially in early stages of embryonic development. Additionally, the observed changes in the development of the circulatory system (pericardial edema) can be related to the overexpression of HEY1 with is related to the control of the cardiovascular system development^3^. Consequently, the possible teratogenic effect observed here may reduce the usefulness of using **RIN-1** in anti-cancer therapy.

## Conclusions

Summing up, our research shows that both activation and inhibition of Notch signalling by low molecular chemical compounds have the potential to limit the growth of HNSCC cells, although this effect may be strongly dependent on cell culture conditions and the tumour microenvironment. Using Notch modulators such as **RIN-1**, **CB-103** and **DAPT** similar anticancer effects may be observed, although the mechanistic reason for these changes may be opposing: Both overexpression and hyperactivation, as well as reduction of Notch activity levels below a physiologically supportive level may result in similar consequences. Notch signalling appears to be tightly controlled in cells that do not harbor any Notch receptor mutations, and any large degree deviation from physiological levels—by either inhibitors or activators—may result in pleiotropic, cytotoxic effects.

### Institutional review board statement

The study was conducted according to the guidelines, and approved by the Institutional Review Board of the Medical University of Lublin.

### ARRIVE guidelines statement

We confirm that this study was designed, performed and reported in accordance with ARRIVE's (Animal Research: Reporting of In vivo Experiments) recommendations (Kilkenny C, Browne WJ, Cuthill IC, Emerson M, Altman DG. Improving bioscience research reporting: the ARRIVE guidelines for reporting animal research. All methods were carried out in accordance with relevant guideline and regulations.

### Supplementary Information

Supplementary Information.

## Data Availability

The data presented in this study are available in presented article and supplementary material. Any additional data related with this study are available on request from the corresponding author.

## References

[1] Siebel C, Lendahl U (2017). Notch signaling in development, tissue homeostasis, and disease. Physiol. Rev..

[2] Zhou, B. *et al.* Notch signaling pathway: architecture, disease, and therapeutics. *Signal Transduct. Target. Ther. 2022 71* **7**, 1–33 (2022). 10.1038/s41392-022-00934-y PMC8948217 35332121

[3] Fischer A, Gessler M (2007). Delta–Notch—and then? Protein interactions and proposed modes of repression by Hes and Hey bHLH factors. Nucleic Acids Res..

[4] Borggrefe T, Oswald F (2009). The Notch signaling pathway: Transcriptional regulation at Notch target genes. Cell. Mol. Life Sci..

[5] Akil A (2021). Notch signaling in vascular endothelial cells, angiogenesis, and tumor progression: An update and prospective. Front. cell Dev. Biol..

[6] Fukusumi T, Califano JA (2018). The NOTCH pathway in head and neck squamous cell carcinoma. J. Dent. Res..

[7] Zhao, Y.-Y., Yu, G.-T., Xiao, T. & Hu, J. The Notch signaling pathway in head and neck squamous cell carcinoma: A meta-analysis. *Adv. Clin. Exp. Med. Off. organ Wroclaw Med. Univ.* **26**, 881–887 (2017). 10.17219/acem/64000 29068587

[8] South, A. P. *et al.* NOTCH1 mutations occur early during cutaneous squamous cell carcinogenesis. *J. Invest. Dermatol.* **134**, (2014). 10.1038/jid.2014.154 PMC4753672 24662767

[9] Yap LF (2015). The opposing roles of NOTCH signalling in head and neck cancer: a mini review. Oral Dis..

[10] Porcheri, C., Meisel, C. T. & Mitsiadis, T. Multifactorial contribution of Notch signaling in head and neck squamous cell carcinoma. *Int. J. Mol. Sci.* **20**, (2019). 10.3390/ijms20061520 PMC6471940 30917608

[11] Porcheri C, Mitsiadis TA (2021). Notch in head and neck cancer. Adv. Exp. Med. Biol..

[12] Hurtado, C. *et al.* Disruption of NOTCH signaling by a small molecule inhibitor of the transcription factor RBPJ. *Sci. Reports 2019 91* **9**, 1–9 (2019). 10.1038/s41598-019-46948-5 PMC6658660 31346210

[13] Lehal R (2020). Pharmacological disruption of the Notch transcription factor complex. Proc. Natl. Acad. Sci. USA.

[14] Lepikhova T (2018). Drug-sensitivity screening and genomic characterization of 45 HPV-negative head and neck carcinoma cell lines for novel biomarkers of drug efficacy. Mol. Cancer Ther..

[15] Kato H (1997). Involvement of RBP-J in biological functions of mouse Notch1 and its derivatives. Development.

[16] Ilagan, M. X. G., Lim, S., Fulbright, M., Piwnica-Worms, D. & Kopan, R. Real-time imaging of Notch activation with a luciferase complementation-based reporter. *Sci. Signal.* **4**, (2011). 10.1126/scisignal.2001656 PMC3387985 21775282

[17] Minoguchi, S. *et al.* RBP-L, a transcription factor related to RBP-Jkappa. *Mol. Cell. Biol.* **17**, (1997). 10.1128/mcb.17.5.2679 PMC232118 9111338

[18] Kałafut, J. *et al.* Optogenetic control of NOTCH1 signalling. 10.1101/2021.09.27.462029.

[19] Jarriault, S. *et al.* Signalling downstream of activated mammalian Notch. *Nature* **377** (1995). 10.1038/377355a0 7566092

[20] Ali Z (2022). Zebrafish patient-derived xenograft models predict lymph node involvement and treatment outcome in non-small cell lung cancer. J. Exp. Clin. Cancer Res..

[21] Panzica-Kelly, J. M. *et al.* Morphological score assignment guidelines for the dechorionated zebrafish teratogenicity assay. *Birth Defects Res. Part B Dev. Reprod. Toxicol.* **89**, 382–395 (2010). 10.1002/bdrb.20260 20836125

[22] Guzman, M. C. De, Chua, P. A. P. & Sedano, F. S. Embryotoxic and teratogenic effects of polyethylene microbeads found in facial wash products in Zebrafish (Danio rerio) using the Fish Embryo Acute Toxicity Test. *bioRxiv* 2020.09.16.299438 (2020). 10.1101/2020.09.16.299438.

[23] Gumbarewicz E (2021). Differential molecular response of larynx cancer cell lines to combined VPA/CDDP treatment. Am. J. Cancer Res..

[24] Härmä V (2014). Quantification of dynamic morphological drug responses in 3D organotypic cell cultures by automated image analysis. PLoS ONE.

[25] Härmä, V. *et al.* A comprehensive panel of three-dimensional models for studies of prostate cancer growth, invasion and drug responses. *PLoS One* **5** (2010). 10.1371/journal.pone.0010431 PMC2862707 20454659

[26] Åkerfelt, M. *et al.* Automated tracking of tumor-stroma morphology in microtissues identifies functional targets within the tumor microenvironment for therapeutic intervention. *Oncotarget* **6** (2015). 10.18632/oncotarget.5046 PMC4745780 26375443

[27] Shah, P. A. *et al.* NOTCH1 Signaling in Head and Neck Squamous Cell Carcinoma. *Cells* vol. 9 (2020). 10.3390/cells9122677 PMC7764697 33322834

[28] Loganathan SK (2020). Rare driver mutations in head and neck squamous cell carcinomas converge on NOTCH signaling. Science.

[29] Pine SR (2018). Rethinking Gamma-secretase Inhibitors for treatment of non–small-cell lung cancer: Is notch the target?. Clin. Cancer Res..

[30] McCaw, T. R. *et al.* Gamma secretase inhibitors in cancer: A current perspective on clinical performance. *Oncologist* vol. **26** (2021). 10.1002/onco.13627 PMC8018325 33284507

[31] Medinger, M. *et al.* CB-103: A novel CSL-NICD inhibitor for the treatment of NOTCH-driven T-cell acute lymphoblastic leukemia: A case report of complete clinical response in a patient with relapsed and refractory T-ALL. *eJHaem* (2022) 10.1002/JHA2.510. 10.1002/jha2.510 PMC9421963 36051082

