# Supplementary material for: Evaluation of the anticancer activity of RIN-1, a Notch signaling modulator, in head and neck squamous cell carcinoma
Source: Sci Rep. 2023 Aug 22;13:13700. doi: 10.1038/s41598-023-39472-0 (PMC10444807; doi:10.1038/s41598-023-39472-0)

**SUPPLEMENTARY DATA**

Anti-tumour activity of Notch signalling modulators RIN-1, CB-103, and DAPT in the Head and Neck Squamous Cell Carcinoma

Arkadiusz Czerwonka ^1,^*, Joanna Kałafut ^1^, Shaoxia Wang ^2^, Alinda Anameric ^1^, Alicja Przybyszewska-Podstawka ^1^, Jesse Mattsson ^2^, Mahtab Karbasian ^2^, Doriane Le Manach ^1^, Mervi Toriseva ^2^ and Matthias Nees ^1^

^1^ Department of Biochemistry and Molecular Biology, Medical University in Lublin, 20-093 Lublin, Poland.
^2^ Institute of Biomedicine, Cancer Research Unit and FICAN West Cancer Centre Laboratory, University of Turku and Turku University Hospital, Turku, Finland.

***** Correspondence: arkadiusz.czerwonka@umlub.pl

**Supplemental Figure I: Expression of key elements of Notch signalling in HNSCC cells**

Compared to primary HNSCC cell lines of the same origin (UT-SCC-24A and UT-SCC-42A), the metastatic/recurrent cell lines (UT-SCC-24B and UT-SCC-42B, respectively) tendentially showed reduced mRNA expression for NOTCH2, NOTCH4, JAG1, MAML2, and DVL3 (**Supplemental Figure IA**). Only DLL3 expression was significantly increased in both advanced, UT-SCC-24B and UT-SCC-42B cells. Additionally, UT-SCC-42B metastatic cells showed a significant increase in NOTCH3, JAG2, DLL1, ADAM17 and RBP-J expression, compared to primary UT-SCC-42A cells.

Western Blot analyses (**Supplemental Figure IB**) demonstrate expression of NOTCH receptors 1-3, ligands (JAG1), and Notch-regulated genes (HES1, HES5) in a HNSCC panel of cell lines. Compared to UT-SCC-24A cell line, lower expression of HES1 and JAG1 and higher expression of NICD3 were observed in UT-SCC-24B. Furthermore, cells lines UT-SCC-24A and UT-SCC-24B showed comparable levels of full length (FL) NOTCH1 and 3, cleaved NOTCH1 and 2 (N1/2-TMICD) and HES5. Interestingly, higher expression of NICD1, NICD3, HES1 and JAG1 was observed in UT-SCC-42B compared to UT-SCC-42A. No striking differences between other proteins tested was observed.


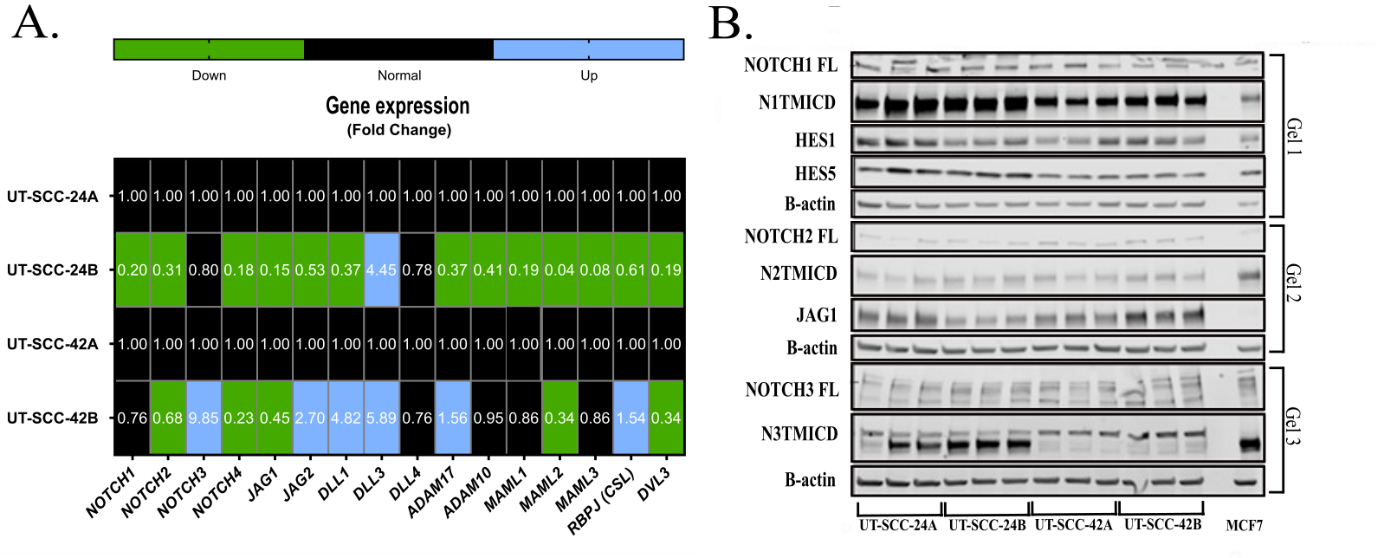


**
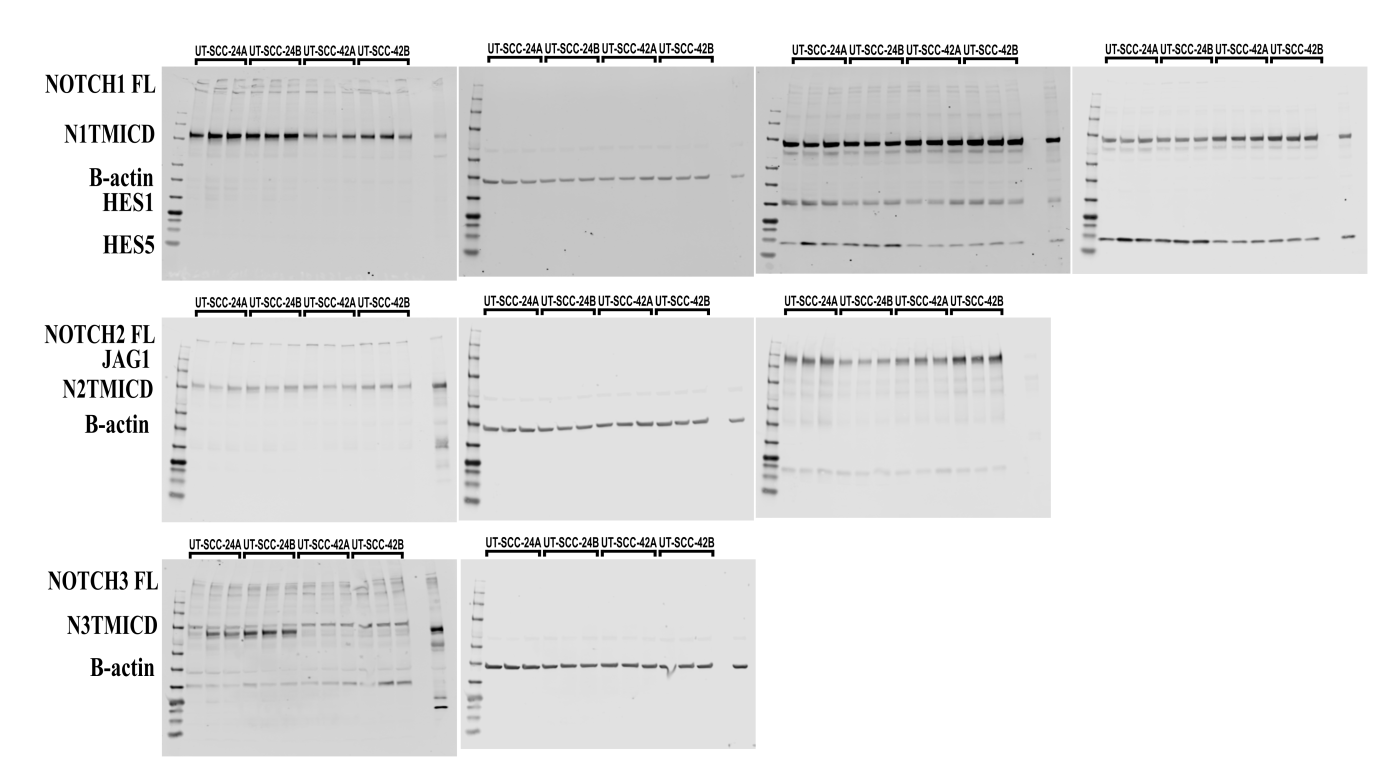
**

**Supplemental Figure I**. The comparison of mRNA expression Notch signalling genes in HNSCC cel lines (UT-SCC-24A vs UT-SCC-24B and UT-SCC-42A vs UT-SCC-42B) (A). Fold change value in the range of 0 - 0.749; was considered as downregulated (green, *p ≤ 0.05) and 1.501 - 10 as upregulated (blue, *p ≤ 0.05), respectively. Expression of Notch signalling-related proteins in non-treated HNSCC cell lines lysates (B; original blots are presented below). The protein level of NOTCH receptors (NOTCH1-3), Jagged ligand (JAG1) and NOTCH target genes (HES1, HES5) are shown. The equal loading amount of protein was verified by internal control (β-actin). Protein lysate of MCF-7 cell line was used as an external control. FL (full length): complete, noncleaved form of NOTCH receptor, N1/2/3-TMICD, (transmembrane and intracellular part of NOTCH1/2/3): cleaved form of NOTCH receptor.

**Supplemental Figure II: The 12xCSL-Luc reporter assay in mock-transfected HEK293 cells (lacking the reporter construct)**


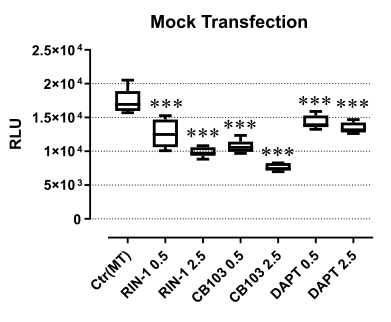


**Supplemental Figure II:** Modulation of Notch signaling by compounds **RIN-1**, **CB-103**, and **DAPT** transfected HEK293 cells with stable expression of a Notch-responsive 12xCSL-Luc reporter system. The relative luminescence units (RLU) level was measured 48 hours after exposure to 0.5 and 2.5 uM of each Notch modulator, respectively.

**Supplemental Figure III: Gene expression heatmap of NOTCH-regulated genes after treatment with NOTCH signalling modulators**

**
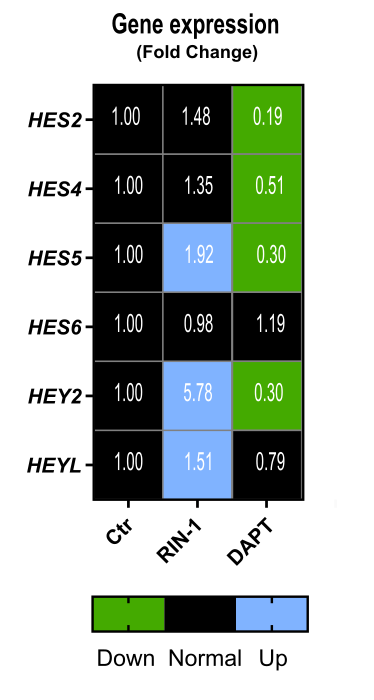
**

**Supplemental Figure III:** The effects of 2.5 uM of each of the 4 Notch modulators on Notch signalling and expression of Notch-regulated downstream genes after 72-h of treatment in UT-SCC-42B cells (2^-∆∆Ct^). The following sequences were used in the research: *HES2* (forward 5'-AACCAGAGCCTGAGCCAGCTTA-3' and reverse 3'-TGCAGGAAGCGCACGGTCATTT-5'), *HES4* (forward 5'-GAGCGCGTATTAACGAGAGCCT-3' and reverse 3'-CTCACGGTCATCTCCAGGATGT-5'), *HES5* (forward 5'-TCCTGGAGATGGCTGTCAGCTA-3' and reverse 3'-CGTGGAGCGTCAGGAACTGCA-5'), *HES6* (forward 5'-GCTGGAGAACGCCGAAGTGCT-3' and reverse 3'-TGGACACGAACGTGTGCACCTC-5'), *HEY2* (forward 5'-TGAGAAGACTTGTGCCAACTGCT-3' and reverse 3'-CCCTGTTGCCTGAAGCATCTTC-5'), *HEYL* (forward 5'-TGGAGAAAGCCGAGGTCTTGCA-3' and reverse 3'-ACCTGATGACCTCAGTGAGGCA-5'),

**Supplemental Figure IV: Effect of Notch-targeting drugs on the growth of HNSCC cells in 2D monolayer cultures**


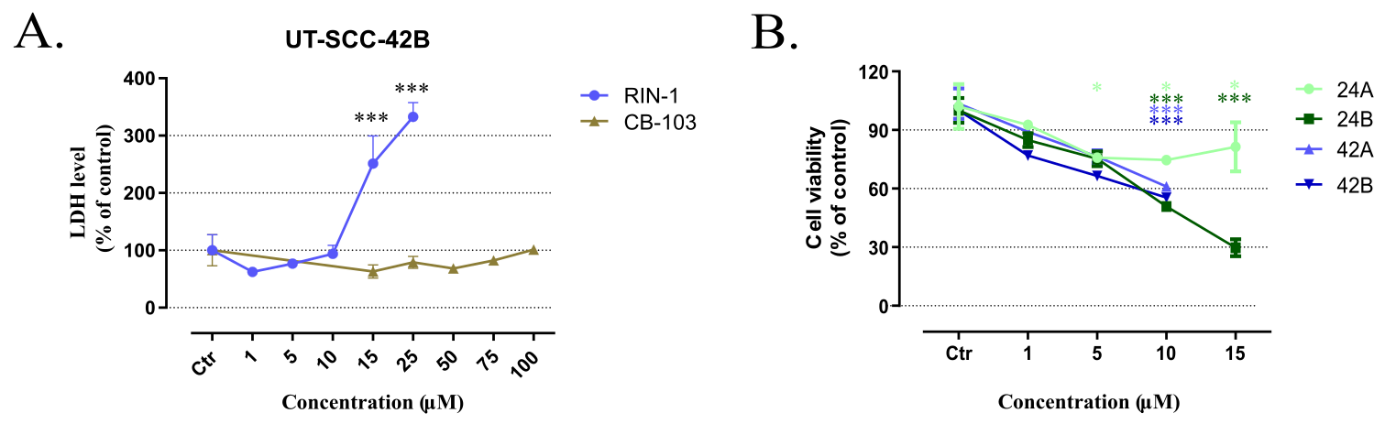


**Supplemental Figure IV:** UT-SCC-42B cells incubated with **RIN-1** (1-25 µM), and **CB-103** (15-100 µM), respectively. The level of cell membrane permeability, which correlates with viability, was measured 24h hours after drug exposure by lactate dehydrogenase (LDH) release assay (A). The viability of RIN-1-treated cells examined with the WST-8 assay after 72-h drug exposure (B).

**Supplemental Figure V: Effect of Notch-targeting drugs on cell motility and migration, chromatin condensation and apoptosis.**


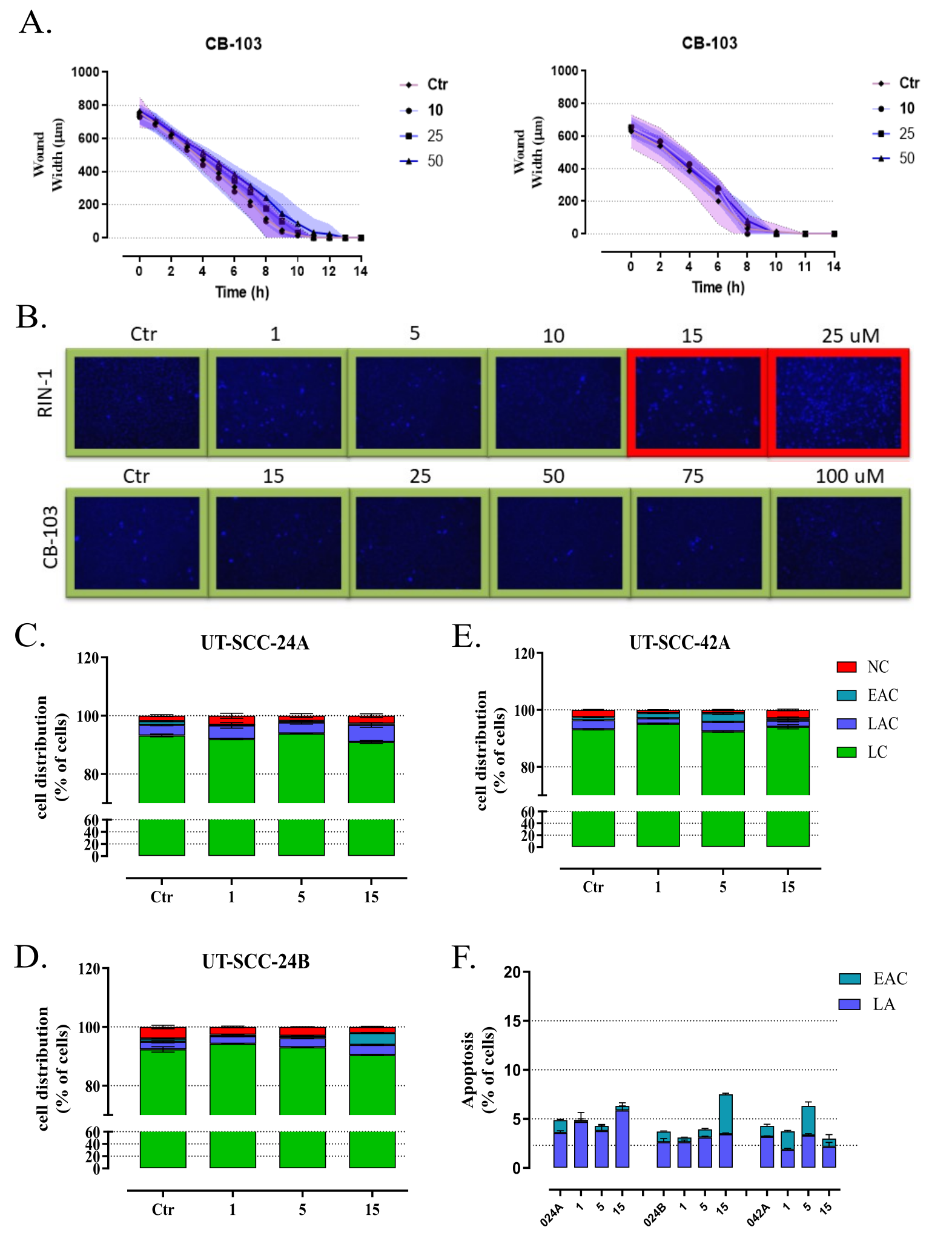


**Supplemental Figure V:** Impact of CB-103 on cell migration or motility of UT-SCC- cells (left panel) and UT-SCC-42B cells (right panel), as assessed by „scratch wound” or ”wound healing” assay (A). Cells were seeded in a 96-well plate and allowed to reach full confluence. Next, after making the wound (using the “woundmaker device”), cells were treated with Notch modulators and analyzed in the IncuCyte for life cell imager for wound closure, using real-time monitoring. A graphical representation of the reduction in wound width (μm) over time during the treatment with Notch modulator is shown. Influence of Notch signalling modulators on chromatin condensation and formation of apoptotic bodies (B). UT-SCC-42B cells were incubated with RIN-1 (1 -25 uM), and CB-103 (15 - 100 uM), respectively. Representative pictures of nuclei in UT-SCC-42B cells, stained with Hoechst 33342 dye after 48h exposure to Notch modulators obtained by the EVOS Cell Imaging Systems. Chromatin condensation is visible as intense blue glow. Green boxes: chromatin condensation on control level; red boxes: increased chromatin condensation. The percentage of necrotic (Annexin V^−^/PI^+^), early apoptotic (Annexin V^+^/PI^−^), late apoptotic (Annexin V^+^/PI^+^) and viable cells (Annexin V^−^/PI^−^) for the HNSCC cell lines (C-E). Dead and dying cells were assessed by Annexin V and Propidium Iodide (PI) staining assay. The total level of apoptosis in each cell line is shown (F). Control (untreated cells) described as 0_24A_, 0_24B_, and 0_42A_ for UT-SCC-24A, UT-SCC-24B, UT-SCC-42A, respectively.

**Supplemental Figure VI - The effect of Notch signalling pathway modulators on cell growth in non-adherent conditions and HNSCC organoids, formed in Matrigel**


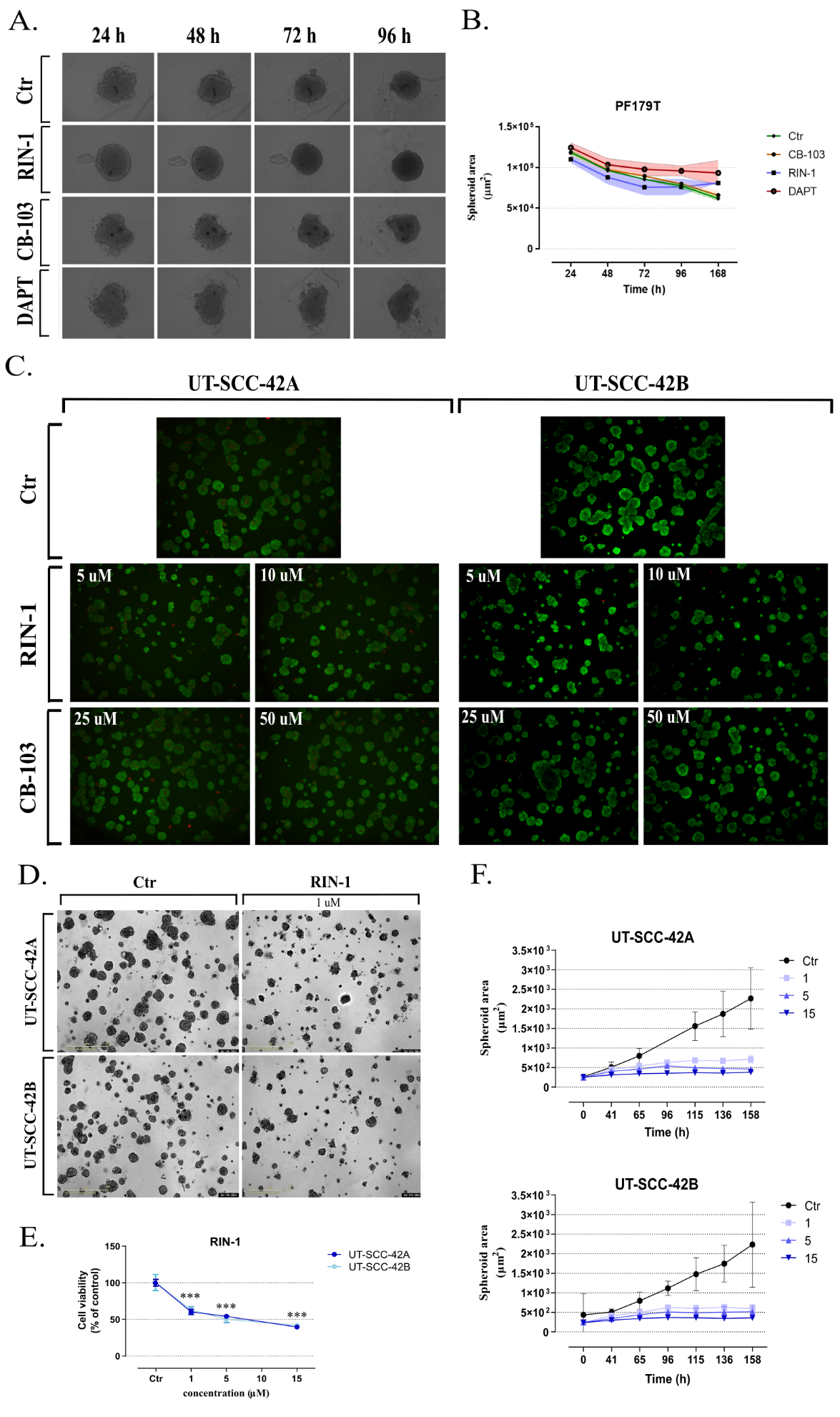


**Supplemental Figure VI:** The effect of Notch signalling modulators on cell growth of PF179T cancer-associated fibroblasts (CAFs) in non-adherent conditions (A). After seeding, cells were immediately treated with Notch modulators and observed for 96 hours. Representative photography of PF179T spheroids after exposure to 2.5 uM of Notch modulators. Graphs of the average size of drug exposed PF179T spheroids (mean ± SD; μm^2^) over time (B). Impact of Notch-pathway modulators on mature, well-differentiated and polarized tumor organoids (C). At the endpoint of 3D experiments, multicellular organoids were double-stained by calcein AM and ethidium homodimer-1 fluorescent (EthD-2) dyes (1: 500 in full medium, incubated at 37 °C for 1h). 3D confocal images were acquired with a Zeiss Axiovert-200M microscope, equipped with Yokogawa CSU22 spinning disc confocal unit using Zeiss Plan-Neofluar 5x objective. Z-stacks (stacked confocal photos) projections were created with SlideBook (Intelligent Imaging Innovations Inc, Denver, CO, USA) software. Impact of Notch modulators on proliferation in 3D cultures of single cells seeded simultaneously with NOTCH modulator drugs: freshly seeded cells were immediately treated by RIN-1. The formation and growth organoids was monitored on the IncuCyte Live Cell Analysis system for 158 hours. Representative images show untreated and drug exposed HNSCC organoids at the endpoints of experiments (D). Cell viability (% of control) at the endpoint of experiments was assessed by WST8 assay (E). Graphs indicate the average size of organoids (mean ± SD; μm^2^) over time for RIN-1 (F).

Supplementary Table 1. Observed IC_50_ values (effective concentrations resulting in inhibition of proliferation by 50% compared to the untreated control cells) for RIN-1, and CB-103. Data are based on MTT assay, obtained after 96-hour exposure of HNSCC cell lines to the four compounds. The IC_50_ values (μM of a tested compound) were calculated from nonlinear regression (log(inhibitor) vs. normalized response-variable slope) according to GraphPad Prism 8.0. software.

|  | **RIN-1** | **CB-103** |
| --- | --- | --- |
| UT-SCC-24A | 14.44 | 180.5 |
| UT-SCC-24B | 15.74 | 465.3 |
| UT-SCC-42A | 3.64 | 52.65 |
| UT-SCC-42B | 2.05 | 80.46 |

**Supplemental Figure VII: Original blots presented in Figure 2.**


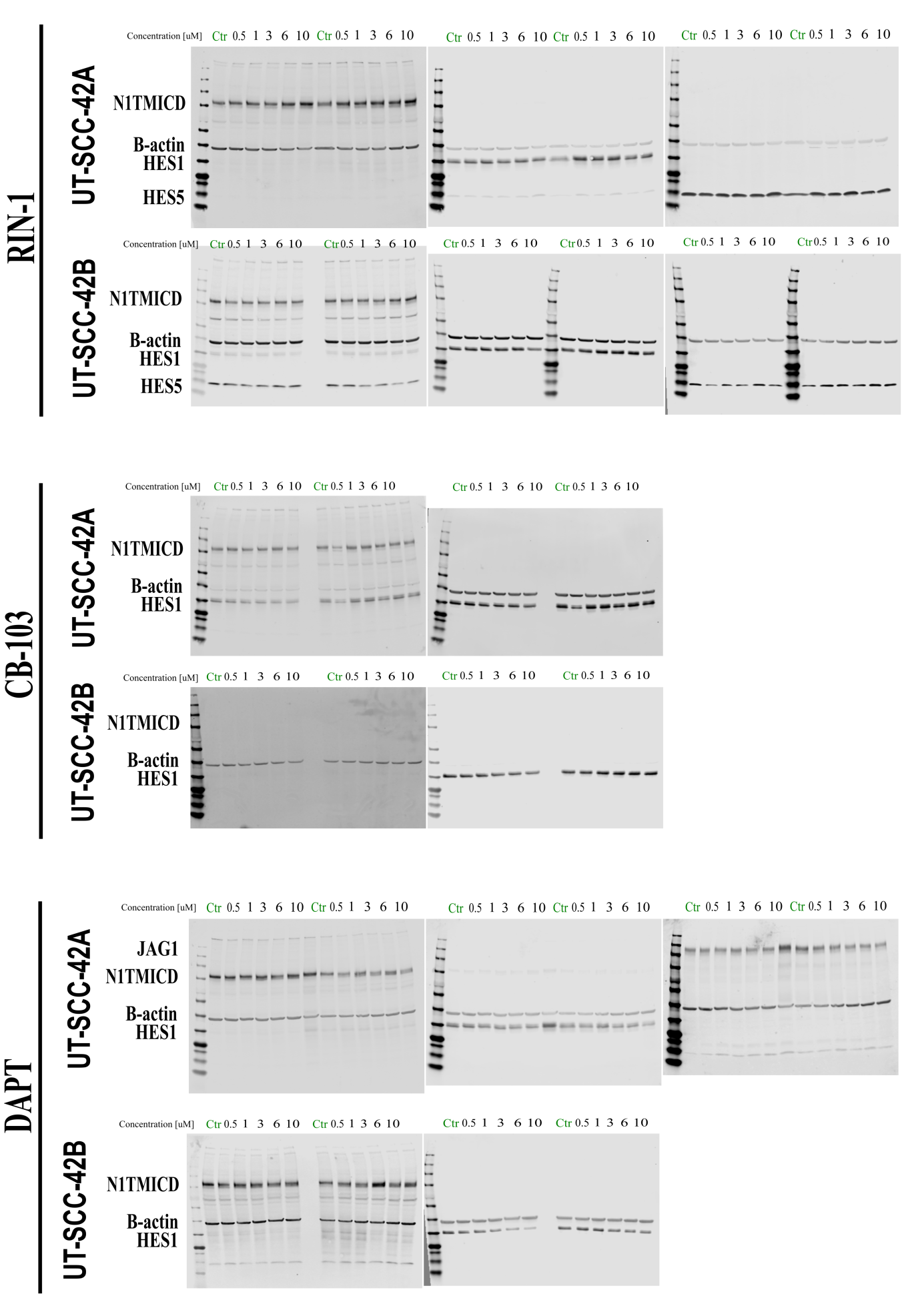

Supplement: Supplementary file 1 — Supplementary Information. [file 41598_2023_39472_MOESM1_ESM.docx]
